# Diagnostic Indices for Epidemiological Assessment of Molar Incisor Hypomineralization: A Systematic Review

**DOI:** 10.1007/s00223-026-01538-2

**Published:** 2026-05-08

**Authors:** Marta Mazur, Artnora Ndokaj, Marta Berdzik-Janecka, Irena Dus-Ilnicka, Roman Ardan, Sylvie Babajko, Katia Jedeon

**Affiliations:** 1 Interdisciplinary Department of Wellbeing, Health and Environmental Sustainability-BeSSA Department, Sapienza University of Rome, Rieti, 02100 Italy; 2 Department of Oral and MaxilloFacial Sciences, Sapienza University, Via Caserta 6, Rome, 00161 Italy; 3 Department and Division of Paediatric Dentistry and Preclinical Dentistry Faculty of Medicine and Dentistry, Wroclaw Medical University, ul. T. Marcinkowskiego 1, Wroclaw, 50-368 Poland; 4 Division of General and Experimental Pathology, Department of Clinical and Experimental Pathology, Wroclaw Medical University, ul. T. Marcinkowskiego 1, Wroclaw, 50-368 Poland; 5 Department of Economic Sciences, Koszalin University of Technology, Koszalin, Poland; 6 Université́ Paris Cité, Université Sorbonne Paris Nord, Institut National de Santé et Recherche Médicale (INSERM), Unité Mixte de Recherche (UMR) 1333 Oral Heath, Montrouge, 92120 France; 7 Department of Restorative Dentistry and Endodontics, Unité de Formation et de Recherche (UFR) Odontologie, Rothschild Hospital, 5 rue Santerre, Paris, 75012 France

**Keywords:** Molar incisor hypomineralization, Epidemiology, Prevalence, Diagnostic criteria, Systematic review

## Abstract

Molar incisor hypomineralization (MIH) is a qualitative developmental defect of enamel and one of the most prevalent oral health conditions in childhood. Over the last two decades, increasing attention has been given to its prevalence and etiology; however, marked heterogeneity in diagnostic criteria and indices has limited the comparability of epidemiological findings. This systematic review aimed to identify and describe the diagnostic indices used for the epidemiological assessment of MIH in the general pediatric population. A systematic literature search was conducted in PubMed, Scopus, and Google Scholar to identify epidemiological studies published between January 2001 and December 2024 reporting MIH prevalence in children. Study selection, data extraction, and methodological quality assessment were performed independently by two reviewers in accordance with PRISMA guidelines. The review protocol was registered in PROSPERO (CRD42022345224). A total of 198 articles were included, corresponding to 201 independent epidemiological studies, as three articles reported results from two distinct study populations. The European Academy of Paediatric Dentistry (EAPD) criteria were used in 171 studies (75.7%), representing the most widely adopted diagnostic index. Other indices, including the modified Developmental Defects of Enamel index (mDDE), Mathu-Muju and Wright, Wetzel and Reckel, MIH Severity Scoring System (MIH-SSS), the MIH Treatment Need Index (MIH TNI) criteria, and FDI criteria, were used infrequently. Studies using EAPD criteria accounted for the largest cumulative sample size and showed a descriptively aggregated MIH prevalence of 12.9%, whereas prevalence estimates varied across alternative diagnostic indices. In conclusion, the lack of uniform diagnostic standards and nomenclature hampers global surveillance and limits the comparability of prevalence data. The adoption of MIH-specific diagnostic criteria, supported by adequate examiner calibration and transparent reporting, is essential to improve the quality, consistency, and interpretability of future epidemiological research.

**Supplementary Information:**

The online version contains supplementary material available at 10.1007/s00223-026-01538-2.

## Introduction

Molar incisor hypomineralization (MIH) is a qualitative developmental defect of dental enamel affecting one to four first permanent molars and frequently associated permanent incisors [1]. Clinically, MIH is characterized by demarcated enamel opacities, post-eruptive enamel breakdown, hypersensitivity, and an increased susceptibility to caries and restorative failure [2]. These features often result in complex clinical management and may significantly impair children’s oral health–related quality of life [3].

Over the past two decades, MIH has emerged as one of the most discussed and clinically relevant dental conditions in developmental age [4]. Owing to its high global prevalence, early onset, and long-term consequences for oral health, MIH is currently considered among the most impactful pediatric dental conditions worldwide [5]. The growing number of epidemiological studies reflects increasing awareness of MIH as a public health concern; however, substantial challenges remain in interpreting and comparing epidemiological data.

One of the main limitations in MIH research is the lack of uniformity in nomenclature, diagnostic definitions, and assessment criteria [6]. Different diagnostic indices and classification systems have been adopted across population-based studies, often with varying thresholds for case definition and severity assessment [6]. This heterogeneity may contribute to wide variability in reported prevalence estimates and hampers meaningful comparisons across studies, geographic areas, and time periods.

Given the central role of epidemiological evidence in understanding disease burden and informing prevention and policy strategies, clarity and consistency in diagnostic approaches are essential. Therefore, to provide an overview of current practices and to improve transparency in MIH epidemiological research, the present systematic review of the literature was conducted. The primary objective was to identify and describe the diagnostic indices used in epidemiological studies on MIH, with particular attention to their frequency of use and application in population-based investigations.

To our knowledge, no previous systematic review has specifically examined the global distribution and methodological use of diagnostic indices in epidemiological studies on MIH.

## Materials and Methods

This systematic review was conducted in accordance with the PRISMA (Preferred Reporting Items for Systematic Reviews and Meta-Analyses) [7] guidelines for reporting systematic reviews. The methodological approach was designed to identify, describe, and synthesize the diagnostic indices used in epidemiological studies investigating molar incisor hypomineralization (MIH). No meta-analysis was planned or performed, as the primary objective was descriptive and methodological. The protocol was registered in PROSPERO (CRD42022345224). Due to the exploratory nature of the review, registration occurred after the initial screening phase.

### Eligibility Criteria

The inclusion criteria were as follows: (a) in-vivo epidemiological studies; (b) peer-reviewed observational studies, including cross-sectional, cohort, and case–control designs; (c) studies conducted in pediatric populations; (d) studies reporting the presence and/or prevalence of MIH, defined as a hypomineralization defect affecting first permanent molars, with or without involvement of permanent incisors; (e) studies explicitly reporting the diagnostic criteria or index used for MIH assessment; and (f) studies published in English.

Exclusion criteria were: (a) in-vitro or experimental studies; (b) case reports, editorials, narrative reviews, conference abstracts, and letters; (c) studies focusing exclusively on enamel defects other than MIH or not allowing extraction of MIH-specific data; (d) studies limited to primary dentition; and (e) systematic reviews and meta-analyses.

### Search Strategy and Study Selection

Three electronic databases—PubMed, Scopus and Google Scholar—were systematically searched to identify epidemiological studies reporting the prevalence of MIH in the general pediatric population. The search covered the period from January 2001 to December 2024, corresponding to the introduction and progressive adoption of the MIH definition.

The search strategy combined free-text terms and MeSH terms related to MIH and epidemiology and was adapted for each database:

(i) PubMed: (“Molar Incisor Hypomineralization“[Title/Abstract] OR “MIH“[Title/Abstract]) AND (prevalence[Title/Abstract] OR epidemiolog*[Title/Abstract] OR survey*[Title/Abstract] OR “cross-sectional“[Title/Abstract]) AND (“Child“[MeSH] OR “Adolescent“[MeSH] OR child* OR pediatric* OR paediatric* OR schoolchild*)
(ii) Scopus: “molar incisor hypomineralization” OR MIH AND prevalence OR epidemiology OR epidemiological OR survey OR “cross sectional” AND child OR children OR pediatric OR paediatric OR adolescent.
(iii) Google Scholar: Due to its limited capability to manage complex Boolean operators, a simplified free-text search strategy was used: “molar incisor hypomineralization” AND prevalence OR epidemiology AND “pediatric OR paediatric “.

**Fig. 1:**
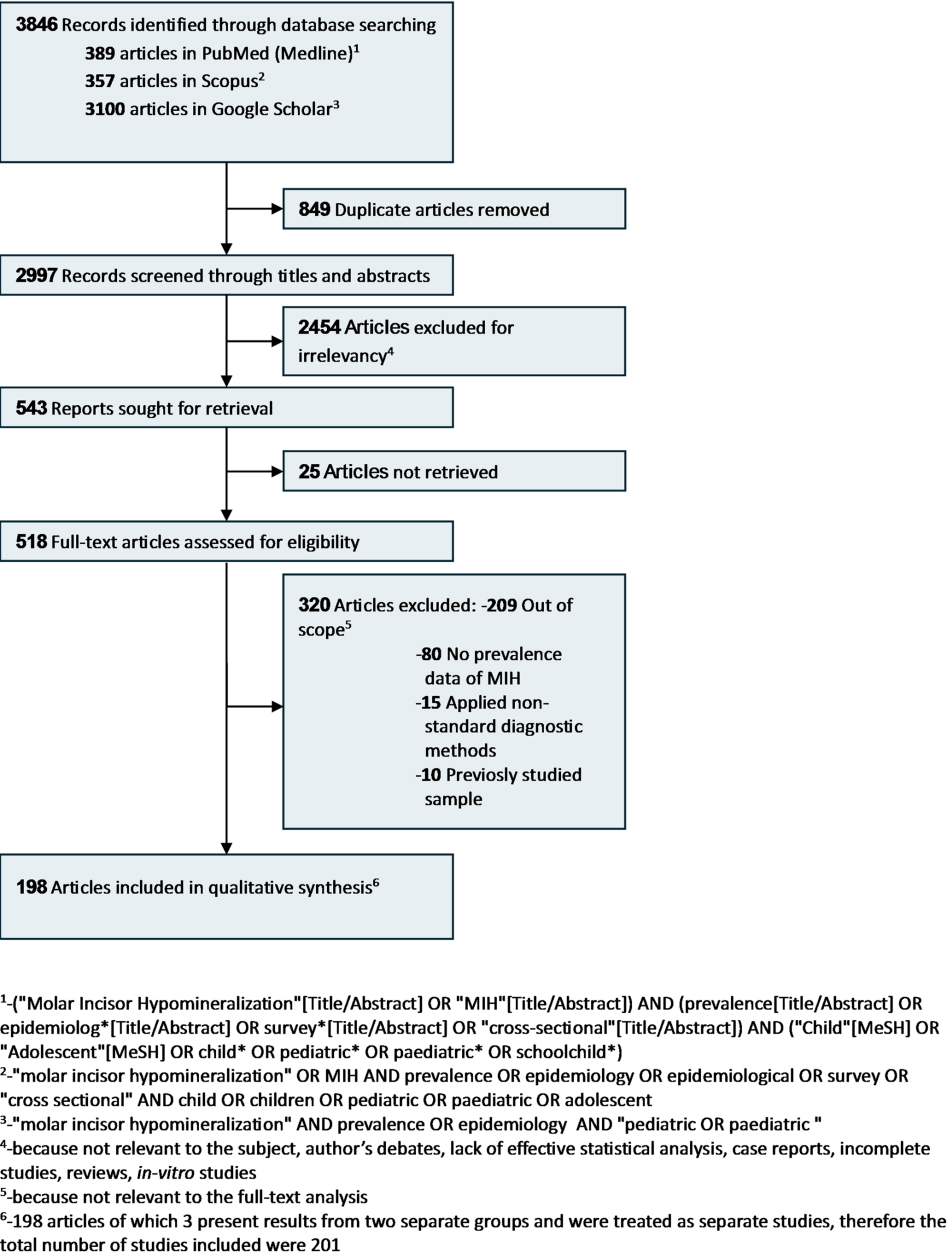
The flow diagram of the search

Searches were conducted independently by two reviewers (MM and AN). Full search strategy is shown in Fig. 1.

### Data Extraction

Data extraction was performed independently by two authors (MM and AN) using a standardized and piloted data extraction form. Extracted information included bibliographic details and methodological characteristics relevant to the objectives of the review. Extracted data were cross-checked for accuracy, and disagreements were resolved by consensus.

### Data Items

The following data items were collected from each included study: year of publication, country, study setting (school-based, hospital-based, or community-based), study design, total sample size, number of participants diagnosed with MIH, reported prevalence, and the diagnostic index or criteria used for MIH assessment. When reported, information on MIH severity classification and examiner calibration was also recorded. (Table S1)

### Methodological Quality Assessment

The methodological quality of included observational studies was assessed using the Newcastle–Ottawa Scale adapted for cross-sectional studies [8]. This tool evaluates study quality based on selection of participants, comparability of study groups, and outcome assessment. Quality scores were calculated for descriptive purposes and were not used as exclusion criteria.

## Results

### Study Selection

The search strategy identified 3,846 records published between 2001 and 2024 (389 from PubMed, 357 from Scopus, and 3,100 from Google Scholar). After removal of 849 duplicates, 2,997 records were screened based on titles and abstracts. Of these, 2,454 were excluded and 25 reports could not be retrieved. A total of 518 full-text articles were assessed for eligibility. After full-text evaluation, 320 studies were excluded (209 out of scope, 80 not reporting MIH prevalence data, 15 applying non-standard diagnostic methods, and 10 based on previously studied samples). Finally, 198 articles were included. As three articles [9–11] reported results from two independent study populations, they were considered as separate studies, resulting in a total of 201 included studies (Fig. 1).

The characteristics of the included studies are summarized in Supplementary Table S1.

### Characteristics of Included Studies

The included studies covered a wide geographic distribution and were conducted across multiple continents. Most investigations were school-based epidemiological surveys involving children in the mixed dentition stage. Sample sizes varied considerably across studies, contributing to a cumulative sample of more than 178,000 examined participants in studies applying the most frequently used diagnostic index.

The main characteristics of the included studies, including country, study setting, sample size, reported MIH prevalence, and diagnostic index used, are presented in Supplementary Table S1.

### Temporal Trends of MIH Epidemiological Studies

**Fig. 2:**
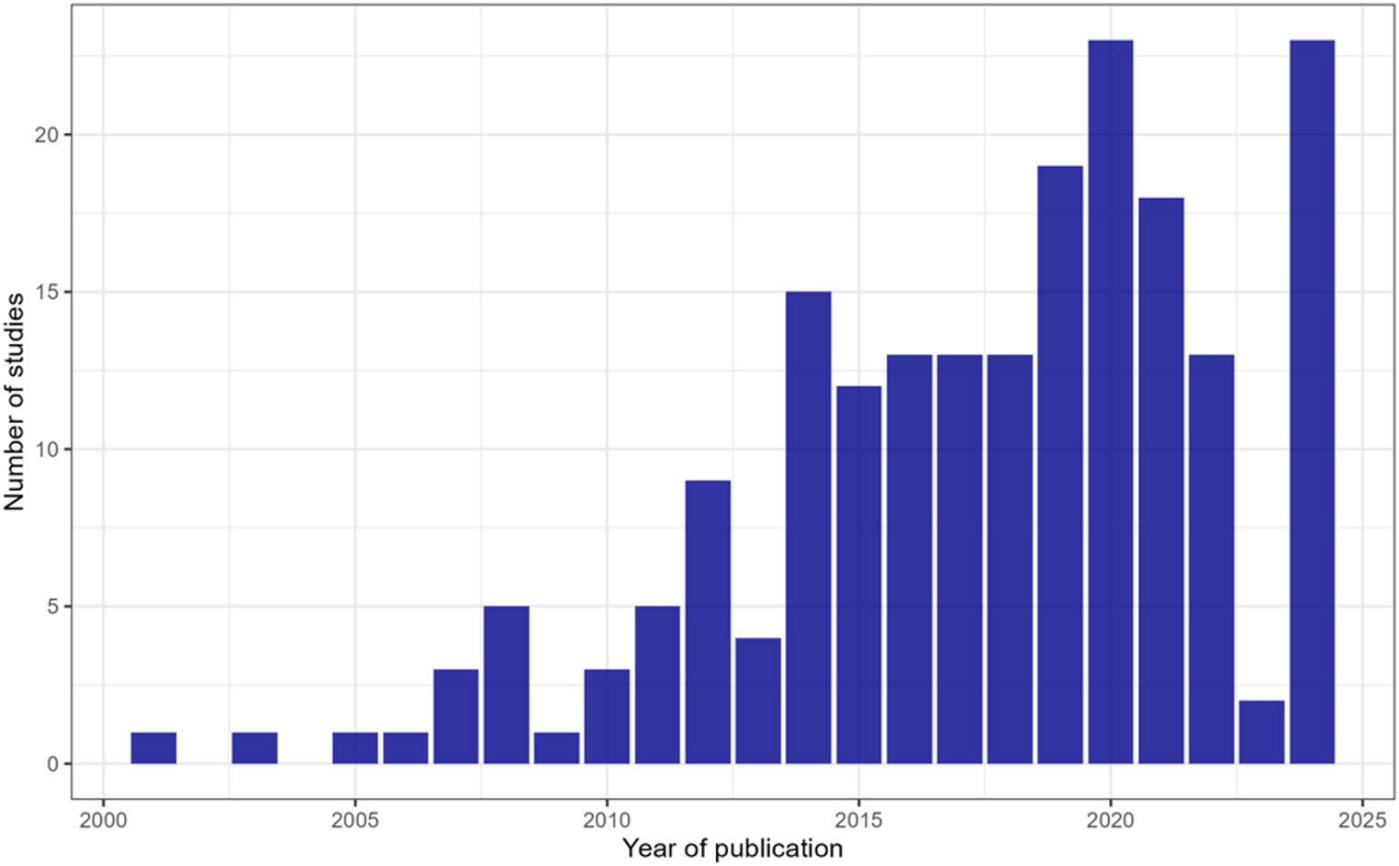
Temporal distribution of epidemiological studies on MIH published between 2001 and 2024. The histogram shows the number of studies per year, illustrating the progressive increase in scientific interest over time, with a marked rise in publications from the mid-2010s onward

The number of epidemiological studies investigating MIH increased progressively over time. A marked rise in scientific output was observed after 2014, reflecting growing interest in MIH as a global oral health concern. The highest number of publications was recorded in 2020 and 2024, each accounting for 23 studies (Fig. 2).

### Distribution of Diagnostic Indices

A clear predominance of the European Academy of Paediatric Dentistry (EAPD) criteria was observed among the included studies. Overall, 171 studies (75.7%) employed the EAPD diagnostic criteria, making it by far the most frequently used index in MIH epidemiological research.

**Fig. 3:**
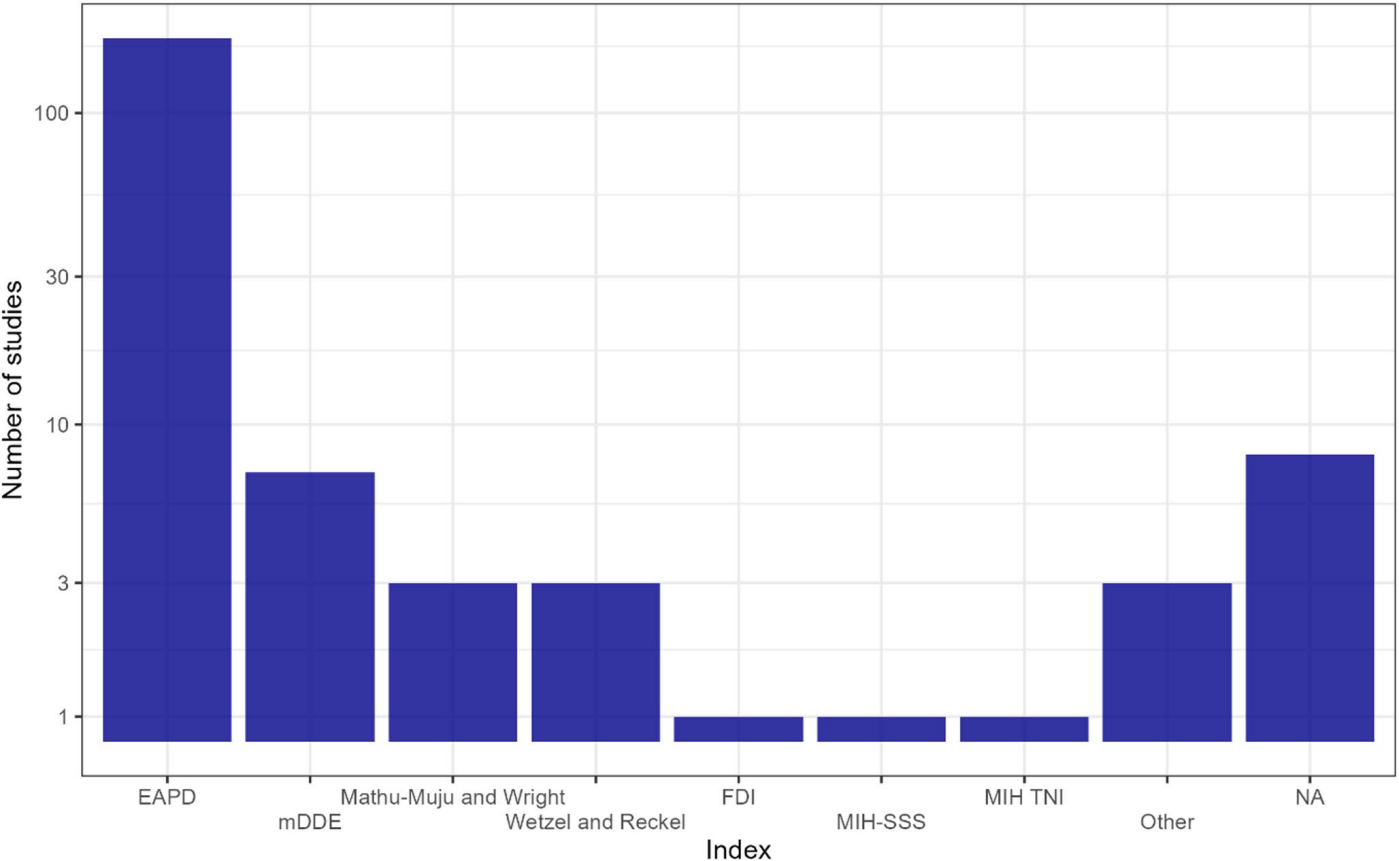
Distribution of diagnostic indices used in epidemiological studies assessing the prevalence of MIH in the pediatric population. The figure illustrates the number of studies adopting each diagnostic index, highlighting the predominant use of the European Academy of Paediatric Dentistry (EAPD) criteria compared with other indices, including mDDE, Mathu-Muju and Wright, Wetzel and Reckel, MIH-SSS, MIH TNI, FDI, and studies in which the diagnostic index was not specified (NA)

Other diagnostic indices were used far less frequently. The modified Developmental Defects of Enamel index (mDDE) was applied in 7 studies, while the Mathu-Muju and Wright criteria and the Wetzel and Reckel classification were each used in 3 studies. Additional diagnostic systems, including the FDI criteria, the MIH Severity Scoring System (MIH-SSS), and the MIH Treatment Need Index (MIH TNI) criteria, were used only once each. In 8 studies, the diagnostic index was not clearly specified (Fig. 3).

### MIH Prevalence According to Diagnostic Indices

Overall and diagnostic indices subgroup prevalence was calculated as the cumulative number of MIH cases divided by the cumulative sample size.

Studies using EAPD criteria accounted for the largest cumulative sample size (178,404 participants). The descriptively aggregated prevalence within this subgroup was 12.9%, corresponding to 23,073 participants diagnosed with MIH. Prevalence estimates varied across diagnostic indices, ranging from 3.8% in studies in which the diagnostic index was not specified to 15.0% in the single study applying the FDI criteria. A detailed summary of the included studies, sample size, number of MIH cases, prevalence estimates, and methodological quality scores according to diagnostic index is presented in Table 1. The ‘Other’ category includes studies using diagnostic approaches not corresponding to established or widely adopted indices, while the mean methodological quality score represents the average Newcastle–Ottawa Scale (NOS) score calculated across studies within each diagnostic category.

**Table 1:** Distribution of epidemiological studies on molar incisor hypomineralization (MIH) according to the diagnostic index applied. The table reports, for each diagnostic criterion, the number of included studies, study setting (hospital- or school-based), total sample size, number of participants diagnosed with MIH, overall MIH prevalence, and mean methodological quality score

Criteria	Studies (*n*.)	Hospital setting	School setting	Total sample	Subjects with MIH	Prevalence (%)	Mean quality
EAPD	171	44	127	178,404	23,073	12.9	7.99
mDDE	7	1	6	10,121	1066	10.5	7.86
Mathu-Maju and Wright	3	1	2	2348	309	13.2	8.33
Wetzel and Reckel	3	0	3	4285	378	8.8	8.33
FDI	1	1	0	233	35	15.0	7.00
MIH-SSS	1	1	0	1405	112	8.0	7.00
MIH TNI	1	0	1	1252	185	14.8	9.00
Other	3	1	2	27,138	2047	7.5	7.00
NA	8	5	3	18,479	697	3.8	6.12

### Geographic Distribution of Studies

**Fig. 4:**
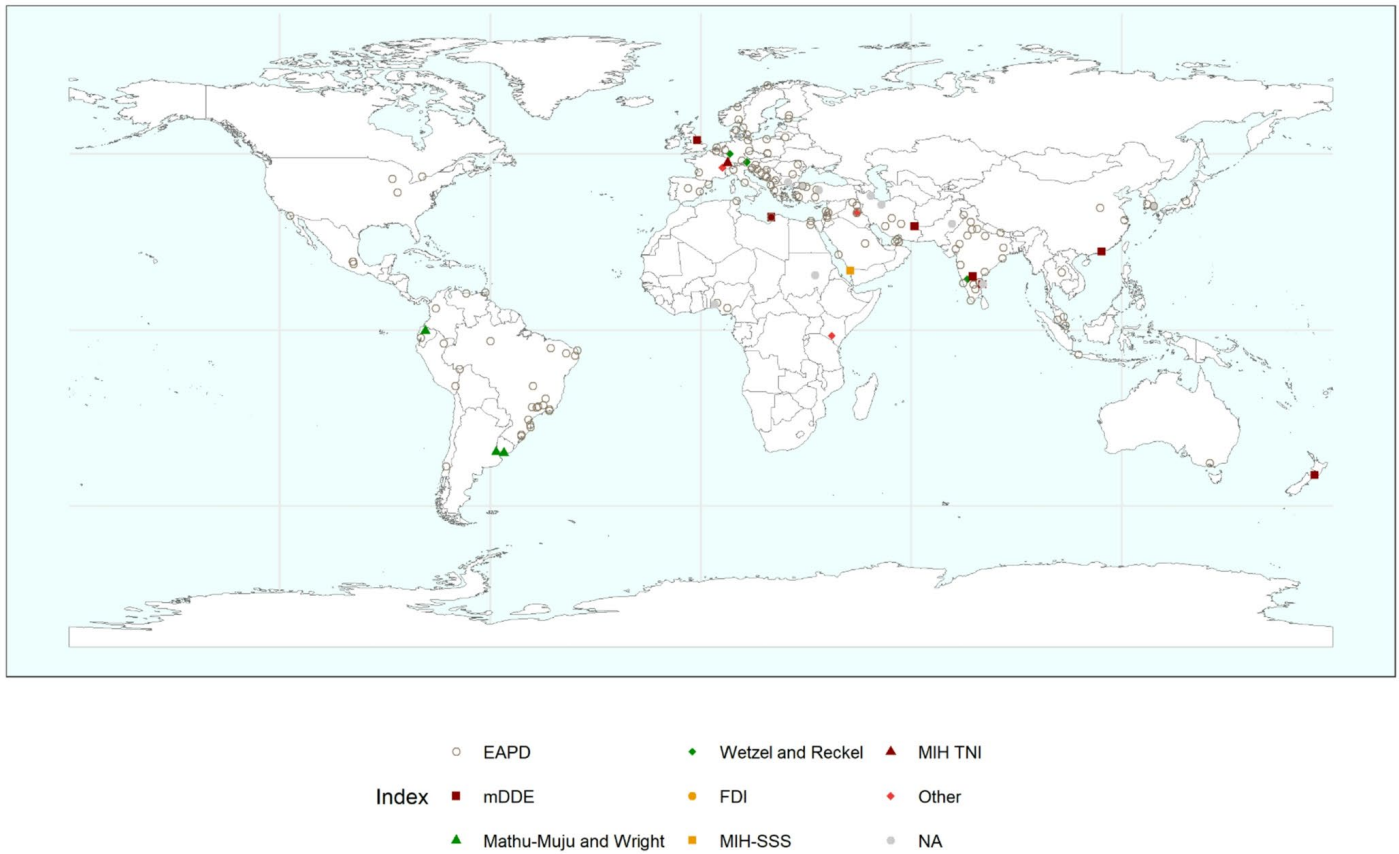
Global geographic distribution of epidemiological studies on molar incisor hypomineralization (MIH) according to the diagnostic index applied. Each point represents the location of a study included in the review and is color-coded by the diagnostic index used (EAPD, mDDE, Mathu-Muju and Wright, Wetzel and Reckel, MIH-SSS, MIH TNI, FDI, Other, or not specified [NA]), illustrating the worldwide predominance of EAPD criteria and the heterogeneous adoption of alternative indices across regions

The included epidemiological investigations were conducted worldwide, covering countries from all inhabited continents. The highest number of studies originated from India and Brazil, reflecting the strong research activity on MIH in these regions. The geographic distribution of the included studies according to the diagnostic index applied is illustrated in Fig. 4.

A comprehensive summary of the distribution of epidemiological studies according to the diagnostic index applied, including sample size, number of MIH cases, prevalence estimates, and methodological quality scores, is presented in Table 1.

### Methodological Quality Assessment

The methodological quality of the included studies was assessed using the Newcastle–Ottawa Scale adapted for cross-sectional studies (Table S2).

Most studies achieved high quality scores ranging from 7 to 9 and were classified as having a low risk of bias. A smaller number of studies scored 6 and were categorized as having a moderate risk of bias, while only one study obtained a score of 5. Overall, the majority of studies performed well across the main NOS domains, including selection of the sample, comparability of study groups, and outcome assessment.

## Discussion

This systematic review provides a comprehensive overview of the diagnostic indices used in epidemiological studies assessing the prevalence of MIH in the global pediatric population. The analysis of 198 articles published between 2001 and 2024 highlights a clear predominance of the European Academy of Paediatric Dentistry (EAPD) criteria, alongside a heterogeneous use of alternative diagnostic systems. This variability has important implications for the interpretation, comparability, and external validity of MIH prevalence data.

The EAPD criteria were by far the most frequently adopted diagnostic approach, accounting for more than three quarters of the included studies [3]. Their widespread use likely reflects their clarity, relative simplicity, and specific focus on MIH as a distinct clinical entity. The EAPD criteria provide standardized definitions of demarcated opacities, post-eruptive enamel breakdown, and atypical restorations, making them particularly suitable for population-based surveys. The large cumulative sample size and the consistent prevalence estimates observed in studies using EAPD criteria support their robustness and epidemiological applicability. However, limitations include potential underestimation of mild lesions and inter-examiner variability when calibration is insufficiently reported.

The modified Developmental Defects of Enamel index (mDDE) was used in a limited number of studies. Although mDDE offers a broader framework for recording enamel defects, its lack of specificity for MIH may reduce diagnostic precision [6]. In epidemiological contexts, this can result in misclassification or dilution of MIH prevalence when other enamel defects are concurrently recorded. Consequently, mDDE may be more appropriate for studies exploring enamel defects in general rather than MIH-focused surveillance.

MIH-SSS was applied in only one study, reflecting its limited diffusion in epidemiological research. While this index provides a structured severity-based scoring system, its greater complexity and emphasis on clinical severity may limit feasibility in large-scale field surveys [12]. Similar considerations apply to the Mathu-Maju [13] and Wright criteria and the Wetzel and Reckel [14]classification, both of which were rarely used. These indices were developed in earlier phases of MIH research and contributed to the initial clinical characterization of the condition; however, their limited standardization and reduced international adoption constrain their utility for contemporary epidemiological comparisons.

The MIH Treatment Need Index (MIH TNI) criteria, although used in a single study, showed a high methodological quality score [15]. This suggests potential value in controlled settings; nevertheless, the absence of broader validation and limited use preclude meaningful comparisons with other indices at the population level.

The FDI criteria were also infrequently applied but yielded the highest reported prevalence estimate [16–18]. This finding underscores how diagnostic thresholds and conceptual frameworks can substantially influence epidemiological outcomes. The FDI approach, designed for broader enamel defect assessment, may capture a wider spectrum of lesions, potentially inflating MIH prevalence when compared with MIH-specific indices.

The principal characteristics of the diagnostic indices identified in this review, including their scope, key diagnostic features, and main conceptual differences, are summarized in Supplementary Table S3. These indices differ not only in their diagnostic thresholds—such as the minimum lesion size, inclusion of post-eruptive enamel breakdown, and criteria used to define demarcated opacities—but also in their specificity for MIH and the extent.

Overall, the observed variability in diagnostic indices contributes to heterogeneity in reported MIH prevalence across regions and time. This hampers accurate global surveillance and limits the ability to monitor trends or evaluate preventive strategies. From a public oral health perspective, the lack of standardized diagnostic criteria poses a significant challenge to policy development and resource allocation.

The coexistence of multiple diagnostic indices partly reflects the historical evolution of the field. Early epidemiological investigations often relied on general indices for developmental defects of enamel, such as the DDE index [19], which were not specifically designed for MIH. As understanding of the condition improved, several MIH-specific indices and severity classifications were proposed to better capture the clinical spectrum of the defect and facilitate epidemiological assessment. However, the absence of an internationally accepted reference standard has resulted in the coexistence of multiple diagnostic approaches.

In addition, ongoing debates regarding terminology and conceptual frameworks have contributed to this heterogeneity. Some authors have proposed broader conceptual models of enamel hypomineralization, suggesting the term “molar hypomineralization (MH)” to encompass defects affecting different stages of dentition within a life-course perspective [20, 21]. While these proposals aim to improve etiological interpretation and preventive approaches, they remain under discussion and have not yet been universally adopted in epidemiological research. The debates following the Enamel 11 3D satellite meeting further highlighted the need for greater conceptual clarity and methodological alignment in MIH research.

Several factors may explain why a universally adopted diagnostic framework has not yet been achieved. Diagnostic indices have emerged at different stages of the scientific development of the field and were designed with different objectives, ranging from the general recording of enamel defects to the specific identification of MIH. In addition, methodological variability across epidemiological studies, including differences in examiner training and calibration procedures, may influence diagnostic thresholds and reporting.

Addressing these challenges will likely require coordinated international efforts aimed at harmonizing diagnostic criteria and methodological standards. Possible initiatives include consensus-building processes involving international expert groups, the development of standardized diagnostic protocols, and clearer reporting recommendations for epidemiological studies. Improved examiner training and calibration procedures would further enhance diagnostic reliability in population-based surveys.

Beyond improving the comparability of epidemiological data, the adoption of standardized diagnostic criteria may also have broader implications for clinical practice and public health. Clear and widely accepted diagnostic frameworks could facilitate earlier recognition of MIH in both clinical and community settings and support more consistent identification of affected children. Earlier diagnosis may enable more appropriate preventive and restorative strategies, particularly in cases presenting hypersensitivity or post-eruptive enamel breakdown.

Standardized diagnostic approaches may also facilitate the development of evidence-based clinical guidelines by enabling more reliable comparisons among studies evaluating preventive and therapeutic interventions. In addition, improved diagnostic clarity may increase awareness of MIH among dental professionals, pediatricians, and other healthcare providers involved in child health, thereby promoting earlier referral and improved interdisciplinary collaboration.

Future epidemiological research should prioritize the use of standardized MIH-specific diagnostic criteria and transparent reporting of examiner calibration and severity assessment.

This study has some limitations that should be considered when interpreting the findings. First, although multiple databases were searched, the inclusion of Google Scholar may have introduced limitations in terms of reproducibility due to its dynamic indexing and limited transparency of search algorithms. Second, the descriptive nature of this systematic review, which did not include a meta-analysis, limits the ability to quantitatively compare prevalence estimates across studies. Finally, as with all literature-based analyses, the findings may be influenced by publication bias, as studies with significant or higher prevalence estimates are more likely to be published.

Based on the available evidence, the EAPD criteria currently appear to represent the most suitable reference framework for population-based studies and should be promoted to enhance methodological consistency and comparability in global MIH epidemiological research. Future international consensus initiatives may play a crucial role in harmonizing diagnostic definitions and methodological standards, thereby strengthening the reliability and comparability of global MIH epidemiological research.

## Supplementary Information

Below is the link to the electronic supplementary material.

Supplementary Material 1

Supplementary Material 2

Supplementary Material 3

Supplementary Material 4

Supplementary Material 5
