# Supplementary material for: Diagnostic Indices for Epidemiological Assessment of Molar Incisor Hypomineralization: A Systematic Review
Source: Calcif Tissue Int. 2026 May 8;117(1):80. doi: 10.1007/s00223-026-01538-2 (PMC13156231; doi:10.1007/s00223-026-01538-2)
Supplement: Supplementary file 1 — Supplementary Material 1 [file 223_2026_1538_MOESM1_ESM.docx]

**Supplementary online content**

**Table S1.** Characteristics of the included studies

**Table S2.** Newcastle-Ottawa scale (NOS) for cross-sectional studies

**Table S3.** Main characteristics of diagnostic indices used in epidemiological studies on MIH

**References** in the Supplementary

This supplementary material has been provided by the authors to give readers additional information about their work.

**Table S1** Characteristics of the included studies

| YEAR | AUTHOR | QUALITY ASSESSMENT | COUNTRY | CONTINENT | CITY | SETTING | TOTAL SUBJECTS | TOTAL SUBJECTS SIZE WITH MIH (NR) | CRITERIA OF DIAGNOSIS (EAPD, mDDE or other) |
| --- | --- | --- | --- | --- | --- | --- | --- | --- | --- |
| 2001 | JaÈlevik | 9 | Sweden | Europe | Gòteborg | school screening | 516 | 77 | EAPD |
| 2003 | Dietrich | 8 | Germany | Europe | Dresden | school screening | 2.408 | 535 | EAPD |
| 2005 | Calderara | 9 | Italy | Europe | Lissone | school screening | 227 | 31 | EAPD |
| 2006 | Fteita | 8 | Libya | Africa | Benghazi | school screening | 378 | 11 | mDDE Index |
| 2007 | Muratbegovic | 8 | Bosnia and Herzegovina | Europe | Sarajevo | school screening | 560 | 69 | EAPD |
| 2007 | Jasulaitytò | 9 | LITUANIA | Europe | Kaunas | school screening | 1.277 | 124 | EAPD |
| 2007 | Preusser | 9 | Germany | Europe | Hesse | school screening | 1.002 | 59 | Wetzel and Reckel |
| 2008 | Cho | 7 | China | Asia | Hong Kong | school screening and hospital setting | 2.635 | 73 | mDDE |
| 2008 | Kemoli | 7 | Kenya | Africa | Matungulu, Kangundo | school screening | 3.591 | 493 | Criteria developed by the author |
| 2008 | Kukleva | 7 | Bulgaria | Europe | Plovdiv | school screening | 2.960 | 106 |  |
| 2008 | Kuscu | 7 | Turkey | Europe | Tausancil | school screening | 153 | 14 | EAPD |
| 2008 | Lydiakis | 9 | Greece | Europe | Athens | hospital setting | 3.518 | 360 | EAPD |
| 2009 | Mahoney | 6 | New Zealand |  | Wainuiomata | school screening | 850 | 78 | mDDE index |
| 2010 | Hasenauer | 7 | Austria | Europe | Tyrol and Salzburg | school screening | 1.283 | 140 | Wetzel and Reckel |
| 2010 | Shin | 5 | Sud Corea | Asia | Busan, Ulsan | school screening | 1.344 | 81 |  |
| 2010 | Da Costa Silva | 9 | Brazil | South America | Bothelos, Minas Gerais | school screening | 918 | 182 | EAPD |
| 2011 | Zawaideh | 9 | Jordan | Asia | Aman, Irbuk, Al-Karak | school screening | 3.241 | 570 | EAPD |
| 2011 | Broga ̊rdh-Roth | 7 | Sweden | Europe | Malmo | hospital setting | 82 | 13 | EAPD |
| 2011 | Martinez Gomez | 7 | Spain | Europe | Barcelona | hospital setting | 505 | 90 | EAPD |
| 2011 | Jans | 6 | Chile | South America | La Frontera | hospital setting | 334 | 56 | EAPD |
| 2011 | Ghanim | 9 | Iraq | Asia | Mosul | school screening | 823 | 153 | EAPD |
| 2012 | Ahmadi | 7 | Iran | Asia | Zahedan | school screening | 433 | 55 | DDE index |
| 2012 | Kühnisch | 9 | Germany | Europe | Munich | hospital setting | 693 | 102 | EAPD |
| 2012 | Elfrink | 9 | Netherlands | Europe | Rotterdam | school screening | 2.327 | 203 | EAPD |
| 2012 | Păsăreanu | 7 | Romania | Europe | Iasi | school screening | 334 | 48 | EAPD |
| 2012 | Parikh | 8 | India | Asia | Gandhinagar | hospital setting | 1.366 | 126 | EAPD |
| 2012 | Ghanim | 9 | Iran | Asia | Shiraz | school screening | 810 | 164 | EAPD |
| 2012 | Heitmuller | 8 | Germany | Europe | Munich | hospital setting | 693 | 167 | EAPD |
| 2012 | Ali Salih | 6 | Iraq | Asia | Baghdad | hospital setting | 227 | 15 | EDI (Enamel Defect index) |
| 2012 | Biondi | 9 | Argentina | South America | Buenos-Aires | hospital setting | 512 | 33 | Mathu-Muju and Wright |
|  |  |  | Uruguay | South America | La Republica | hospital setting | 463 | 33 | Mathu-Muju and Wright |
| 2013 | Durmus | 6 | Turkey | Europe | Istanbul | hospital setting | 228 | 54 | EAPD |
| 2013 | Sonmez | 9 | Turkey | Europe | Ankara | school screening | 4.049 | 308 | EAPD |
| 2013 | Jeremias | 9 | Brazil | South America | Araraquara | school screening | 1.157 | 142 | EAPD |
| 2013 | Grošelj | 7 | Slovenia | Europe | Lubiana | school screening | 308 | 66 | EAPD |
| 2014 | Noori | 9 | Iraq | Asia | Sulaimani City | school screening | 2.346 | 427 | EAPD |
| 2014 | Jankovic | 7 | Bosnia and Herzegovina | Europe | Foca | school screening | 141 | 18 | EAPD |
| 2014 | Pitiphat | 7 | Thailand | Asia | Khon Kaen | school screening | 484 | 95 | EAPD |
| 2014 | Pitiphat | 7 | Thailand | Asia | Khon Kaen | school screening | 282 | 78 | EAPD |
| 2014 | Woullett | 9 | Finland | Europe | Helsinki | school screening | 818 | 140 | EAPD |
| 2014 | Shrestha | 9 | Nepal | Asia | Kavre | school screening | 747 | 102 | EAPD |
| 2014 | Lopez Jordi | 9 | Argentina, Uruguay | South America | Buenos Aires, Montevideo | school screening | 1.716 | 252 | Mathu-Maju and Wright |
| 2014 | Petrou | 9 | Germany | Europe | West: Dusseldorf, East: Greifswald, North: Hamburg/Bezirk Eimsbuttel, South: Heidelberg,Rhein-Neckar-Kreis | school screening | 2.395 | 242 | EAPD |
| 2014 | Garcia-Margarit | 9 | Spain | Europe | Valencia | school screening | 840 | 183 | EAPD |
| 2014 | Ng | 9 | Singapore | Asia | Singapore | school screening | 1.083 | 135 | EAPD |
| 2014 | Pruneda | 7 | Mexico | North America | Mexico City | school screening | 433 | 60 | EAPD |
| 2014 | Allazzam | 7 | Saudi Arabia | Asia | Jedah | hospital setting | 267 | 23 | EAPD |
| 2014 | Bhaskar | 9 | India | Asia | Udaipur | hospital setting | 1.173 | 111 | EAPD |
| 2014 | Haidary | 9 | Germany | Europe | Greifswald | school screening | 440 | 19 | EAPD |
|  |  |  | United Arab Emirates (UAE) | Asia | Dubai | school screening | 779 | 59 | EAPD |
| 2014 | Mittal | 9 | India | India | Chandigarh | school screening | 1.792 | 113 | EAPD |
| 2015 | Temilola | 9 | Nigeria | Africa | Osun State | school screening | 1.169 | 48 | EAPD |
| 2015 | Lima | 6 | Brazil | South America | Teresina | school screening | 594 | 109 | EAPD |
| 2015 | Balmer | 9 | England | Europe | London | school screening | 3.233 | 517 | mDDE |
| 2015 | Oyedele | 6 | Nigeria | Africa | Ife-Ife | school screening | 469 | 83 | EAPD |
| 2015 | Krishnan | 8 | India | Asia | Salem | school screening | 4.989 | 384 | EAPD |
| 2015 | Ofi | 7 | Iraq | Asia | Al-Najaf Governorate | school screening | 532 | 122 | EAPD |
| 2015 | Kirthiga | 9 | India | Asia | Davangere | school screening | 2.000 | 179 | Wetzel and Reckel |
| 2015 | Kairala | 7 | Brazil | South America | Gama | school screening | 168 | 9 | EAPD |
| 2015 | Hanan | 9 | Brazil | South America | Manaus | school screening | 2.062 | 188 | EAPD |
| 2015 | Kevrekidou | 9 | Greece | Europe | Ioannina,Ptolemaida Thessaloniki | school screening | 2.335 | 498 | EAPD |
| 2015 | Tadikonda | 7 | India | Asia | Udupi | school screening | 352 | 95 | EAPD |
| 2015 | Hussein | 6 | Malaysia | Asia | Shah Alam | hospital setting | 154 | 26 | EAPD |
| 2016 | Yannam | 8 | India | Asia | Chennai | school screening | 2.864 | 277 | EAPD |
| 2016 | Tourino | 9 | Brazil | South America | Lavras | school screening | 1.181 | 241 | EAPD |
| 2016 | Hussain | 7 | United Arab Emirates | Asia | Dubai and Hatta | school screening | 342 | 93 | EAPD |
| 2016 | Garot | 9 | France | Europe | Bordeaux | hospital setting | 469 | 75 | EAPD |
| 2016 | Siddaiah | 9 | India | Asia | Bangalore | school screening | 1.004 | 115 | EAPD |
| 2016 | Ullah | 7 | Pakistan | Asia | Multan | hospital setting | 233 | 35 | FDI index |
| 2016 | Schmalfuss | 9 | Norway | Europe | Northern Norway, Tromsø and Balsfjord | school screening | 794 | 110 | EAPD |
| 2016 | Negre-Barber | 7 | Spain | Europe | Valencia | hospital setting | 414 | 100 | EAPD |
| 2016 | Martinovic | 9 | Kosovo | Europe | Mitrovica | hospital setting | 712 | 87 | EAPD |
| 2016 | Andrade | 7 | Brazil | South America | Teresina | hospital setting | 66 | 16 | EAPD |
| 2016 | Mishra | 9 | India | Asia | Utar Pradesh | school screening | 1.369 | 191 | EAPD |
| 2016 | Kim | 9 | South Corea | Asia | Jeonju | school screening | 950 | 67 | EAPD |
| 2016 | Hysi | 8 | Albania | Europe | Tirana | hospital setting | 1.575 | 227 | EAPD |
| 2017 | Quintana | 7 | Ecuador | South America | Rumiñahui | school screening | 120 | 24 | Mathu-Muju and Wright index |
| 2017 | Hong | 9 | Cina | Asia | Suzhou | school screening | 1.145 | 51 | EAPD |
| 2017 | Gurrusquieta | 9 | Mexico | North America | Mexico City | school screaning | 1.156 | 183 | EAPD |
| 2017 | Almaz | 8 | Turkey | Asia | Kırıkkale | hospital setting | 9.173 | 23 |  |
| 2017 | Figueiredo | 9 | Brazil | South America | Brazilia | school screening | 858 | 126 | EAPD |
| 2017 | Sidaly | 7 | Norway | Europe | Oslo | hospital setting | 225 | 11 | EAPD |
| 2017 | Shin | 9 | South Corea | Asia | Yangsan | hospital setting | 1.371 | 189 | EAPD |
| 2017 | Poureslami | 9 | Iran | Asia | Kerman | school screening | 779 | 51 | EAPD |
| 2017 | Priya | 9 | India | Asia | Tirupati | hospital setting and school screening | 1.248 | 49 | EAPD |
| 2017 | Buchraber | 9 | Austria | Europe | Graz | school screening | 1.111 | 78 | EAPD |
| 2017 | Lago | 7 | Brazil | South America | Araraquara/SP | school screening | 545 | 78 | EAPD |
| 2017 | Baakkal | 7 | Turkey | Europe | Istanbul | hospital setting | 461 | 54 |  |
| 2017 | Mulic | 7 | Bosnia and Herzegovina | Europe | Kljuc | school screening | 103 | 12 | EAPD |
| 2018 | Koruyucu | 9 | Turkey | Europe/Asia | Istanbul | school screening | 1.511 | 215 | EAPD |
| 2018 | Hernández | 8 | Spain | Europe | Barcelona | school screening | 705 | 56 | EAPD |
| 2018 | Folayan | 9 | Nigeria | Africa | Ile-Ife and Ibadan | school screening | 853 | 25 | EAPD |
| 2018 | Folayan | 0 | Nigeria | Africa | Ife-Ife | hospital setting | 2.107 | 267 |  |
| 2018 | Saitoh | 9 | Japan | Asia | Different regions of Japan | hospital setting | 4.496 | 892 | EAPD |
| 2018 | Teixeira | 7 | Brazil | South America | Teresina | school screening | 334 | 98 | EAPD |
| 2018 | Gambetta-Tessini | 7 | Australia | Australia | Melbourne | hospital setting | 327 | 48 | EAPD |
| 2018 | Saber | 8 | Egypt | Africa | Cairo | hospital setting | 1.001 | 23 | EAPD |
| 2018 | Rai | 9 | India | Asia | Muradnagar | school screening | 992 | 212 | mDDE |
| 2018 | Samuel | 9 | India | Asia | Tiruchengode | school screening | 4.495 | 236 | EAPD |
| 2018 | Padavala | 6 | India | Asia | Chennai | school screening | 170 | 22 | EAPD |
| 2018 | Dantas-Neta | 7 | Brazil | South America | Teresina | school screening | 744 | 186 | EAPD |
| 2018 | Al-Hammad | 9 | Saudi Arabia | Asia | Riyadh | hospital setting | 924 | 376 | EAPD |
| 2019 | Medina | 7 | Peru | South America | Puno Island | school screening | 270 | 31 | EAPD |
| 2019 | Maldonado | 7 | Peru | South America | San Lorenzo | school screening | 225 | 32 | EAPD |
| 2019 | Irigoyen-Camacho | 7 | Mexico | North America | Mexico city | school screening | 232 | 47 | EAPD |
|  |  |  | Mexico | North America | Mexico city | school screening | 317 | 101 | EAPD |
| 2019 | Praptiwi | 8 | Indonesia | Asia | Bandung City | school screening | 619 | 118 | EAPD |
| 2019 | Ardini | 6 | Malaysia | Asia | Kuantan | hospital setting | 156 | 23 | EAPD |
| 2019 | Menoncin | 9 | Brazil | South America | Curitiba | school screening | 731 | 85 | EAPD |
| 2019 | Glodkowska | 9 | Poland | Europe | Pomeranian Region | school screening | 1.437 | 88 | EAPD |
| 2019 | Santos | 9 | Brazil | South America | Florianopolis | school screening | 1.589 | 151 | EAPD |
| 2019 | Rai | 9 | India | Asia | Virajpet | school screening | 1.600 | 120 | mDDE |
| 2019 | Kılınç | 9 | Turkey | Europe | Izmir city | hospital setting | 1.237 | 142 | EAPD |
| 2019 | Herojit Singh | 9 | India | Asia | Jaipur city | school screening | 434 | 18 | EAPD |
| 2019 | Portella | 9 | Brazil | South America | Curitiba | school screening | 728 | 88 | EAPD |
| 2019 | Reyes | 9 | Brazil | South America | Curitiba | school screening | 731 | 88 | EAPD |
| 2019 | Goswami | 8 | India | Asia | Delhi | hospital setting | 1.026 | 12 | EAPD |
| 2019 | Mejia | 9 | Colombia | South America | Medellin | school screening | 1.075 | 120 | EAPD |
| 2019 | Davenport | 7 | Wisconsin, USA | North America | Milwaukee | school screening | 368 | 36 | EAPD |
| 2019 | Agarwal | 9 | India | Asia | (Pune, Maharashtra - West and east of India) | school screening | 1.080 | 90 | EAPD |
| 2019 | Villanueva Gutiérrez | 7 | Mexico | North America | Naucalpan | school screening | 411 | 166 | EAPD |
| 2020 | Glodkowska | 9 | Poland | Europe | Pomerania | school screening | 2.354 | 212 | EAPD |
| 2020 | Einollahi | 7 | Iran | Asia | Ardebil | school screening | 520 | 125 | EAPD |
| 2020 | Da Silva | 7 | Brazil | South America | Rio de Janeiro | hospital setting | 407 | 59 | EAPD |
| 2020 | Zafar | 6 | Pakistan | Asia | Multan | hospital setting | 300 | 10 |  |
| 2020 | Fernandes | 7 | Brazil | South America | São João do Rio do Peixe, Poço José de Moura, Uiraúna | school screening | 610 | 60 | EAPD |
| 2020 | Hamdan | 9 | Jordan | Asia | Amman | school screening | 1.412 | 186 | EAPD |
| 2020 | Sakly | 8 | Tunisia | Africa | Tunis | school screening | 510 | 181 | EAPD |
| 2020 | Jurlina | 8 | Croatia | Europe | Osijek | school screening | 729 | 95 | EAPD |
| 2020 | Wogelius | 9 | Denmark | Europe | Aalborg | hospital setting | 1.837 | 542 | EAPD |
| 2020 | Sidhu | 7 | Canada | North America | Toronto | hospital setting | 233 | 29 | EAPD |
| 2020 | Fragelli | 9 | Brazil | South America | Araraquara | school screening | 467 | 92 | EAPD |
| 2020 | Tagelsir Ahmed | 7 | Indiana | North America |  | school screening | 337 | 43 | EAPD |
| 2020 | Bahrololoomi | 7 | Iran | Asia | Yazd | school screening | 645 | 154 | EAPD |
| 2020 | Elzein | 6 | Lebanon | Europe | Beirut | school screening | 659 | 176 | EAPD |
| 2020 | Singh | 6 | India | Asia | New Delhi | school screening | 649 | 97 | EAPD |
| 2020 | Ray | 9 | India | Asia | Cuttack, Oshida | school screening | 1.525 | 87 | EAPD |
| 2020 | Emmatty | 9 | India | Asia | Kerala | school screening | 5.318 | 216 | EAPD |
| 2020 | Ganapathi | 8 | India | Asia | Chennai | hospital setting | 1.434 | 14 |  |
| 2020 | Bulani | 9 | India | Asia | Pune | school screening | 1.400 | 103 | EAPD |
| 2020 | Hoyte | 7 | Trinidad and Tobago | North-America | Port of Spain | school screening | 546 | 7 | EAPD |
| 2020 | Tseveenjav | 9 | Finland | Europe | Helsinki | hospital setting | 3.042 | 252 | EAPD |
| 2020 | Gorni Dos Reis | 7 | Brazil | South America | Petropolis | school screening | 450 | 129 | EAPD |
| 2020 | Shojaeepour | 9 | Iran | Asia | Kerman | school screening | 2.507 | 129 | EAPD |
| 2021 | Dourado | 7 | Brazil | South America | Lagoas in S~ao Raimundo Nonato,Piauí | school screening | 251 | 117 | EAPD |
| 2021 | Manzoor | 6 | India | Asia | Kashmir | school screening | 480 | 80 | EAPD |
| 2021 | Alhowaish | 9 | Saudi Arabia | Asia | Riyadh | school screening | 893 | 362 | EAPD |
| 2021 | Bonzanini | 7 | Brazil | South America | Esancia Velha | school screening | 513 | 101 | EAPD |
| 2021 | Arheiam | 9 | Libya | Africa | Benghazi | school screening | 1.045 | 162 | EAPD |
| 2021 | Almuallem | 8 | Saudi Arabia | Asia | Riyadh | school screening | 1.562 | 238 | EAPD |
| 2021 | Rodriguez | 7 | Venezuela | South America | Caracas | hospital setting | 121 | 31 | EAPD |
| 2021 | Padmanabhan | 9 | United Arab Emirates (UAE) | Asia | Ras Al Khaimah | school screening | 1.200 | 254 | EAPD |
| 2021 | Khazaei | 9 | Germany | Europe | Munich | hospital setting | 1.072 | 147 | EAPD |
| 2021 | Rajic | 7 | Croatia | Europe | Zagreb | hospital setting | 144 | 9 | EAPD |
| 2021 | Ravichandra | 9 | India | Asia | Gannavaram Mandal, Krishna District, Andhra Pradesh | school screening | 2.250 | 47 | EAPD |
| 2021 | Hali | 8 | Iran | Asia | Sari | school screening | 700 | 142 |  |
| 2021 | Abdalla | 7 | Sudan | Africa | Khartoun state | school screening | 568 | 114 | EAPD |
| 2021 | Bibin | 6 | India | Asia | Jaipur area (villages: Toda Meena, Basma) | school screening | 490 | 30 | EAPD |
| 2021 | Chavez-Cruz | 7 | Mexico | North America | Tijuana | hospital setting | 68 | 21 | EAPD |
| 2021 | Freitas Fernandes | 7 | Brazil | South America | Campina Grande | school screening | 463 | 50 | EAPD |
| 2021 | Farias | 7 | Brazil | South America | Campina Grande | school screening | 471 | 46 | EAPD |
| 2021 | Mohamed | 7 | Saudi Arabia | Asia | Taif | hospital setting | 577 | 207 | EAPD |
| 2022 | Abdelaziz | 8 | Switzerland | Europe |  | school screening | 23.320 | 1.539 | Software-based scoring index |
|  |  |  |  |  |  |  |  |  |  |
| 2022 | Mariam | 9 | India | India |  | school screening | 3.176 | 372 | EADP |
| 2022 | Khan | 9 | India | Asia | Moradabad | school screening | 2.300 | 91 | EAPD |
| 2022 | El Ghaffar | 7 | Egypt | Africa | Giza | school screening | 371 | 10 | EAPD |
| 2022 | Zhang | 9 | China | Asia | Lankao | school screening | 1.568 | 215 | EAPD |
| 2022 | Børsting | 7 | Norway | Europe | Trondheim and Stavanger | hospital setting | 176 | 55 | EAPD |
| 2022 | Vanhée | 7 | Belgium | Europe | Brussels | school screening | 289 | 54 | EAPD |
| 2022 | Verma | 9 | India | Asia | Lucknow | hospital setting | 5.585 | 427 | EAPD |
| 2019 | Ordonez-Romero | 7 | Ecuador | South America | Guayaquil | school screening | 249 | 23 | EAPD |
| 2022 | Argote Quispe | 7 | Peru | South America | Puno | school screening | 404 | 80 | EAPD |
| 2022 | Nisii | 9 | Italy | Europe | Rome | school screening | 346 | 63 | EAPD |
| 2022 | Quintero | 6 | Colombia | South America | Medellin | school screening | 450 | 102 | EAPD |
| 2022 | Grieshaber | 9 | Switzerland | Europe | Basel-Landschaft | school screening | 1.252 | 185 | MIH TNI index |
| 2022 | Ilczuk-Rypuła | 9 | Poland | Europe | Bwtom | hospital setting | 613 | 38 | EAPD |
| 2023 | Ciocan | 6 | Romania | Europe | Bucharest | school screening | 266 | 38 | EAPD |
| 2023 | Al‐Nerabieah | 9 | Siria | Asia | Damascus City | school screening | 1.138 | 452 | EAPD |
| 2024 | Cots | 8 | Spain | Europe | Barcelona | school screening | 1302 | 161 | EAPD |
| 2024 | Tessari | 9 | Brazil | South America | Itajaí | school screening | 603 | 148 | EAPD |
| 2024 | Chowdhury | 8 | India | Asia | Lucknow | school screening | 800 | 41 | EAPD |
| 2024 | Afzal | 8 | Norvegia | Europe | Oslo | hospital setting | 3013 | 538 | EAPD |
| 2024 | Freitas Fernandes | 9 | Brazil | South America | Campina Grande | school screening | 403 | 54 | EAPD |
| 2024 | Sharma | 9 | India | Asia | Lucknow | school screening | 3030 | 174 | EAPD |
| 2024 | Al Jeghami | 7 | Siria | Asia | Damasco | school screening | 2000 | 137 | EAPD |
| 2024 | Brejawi | 7 | UAE | Asia | Fujairah | school screening | 162 | 63 | EAPD |
| 2024 | Seloglu | 7 | Turkey | Asia | Konya | hospital setting | 208 | 104 | EAPD |
| 2024 | Abdulla | 7 | Iraq | Asia | Baquba | school screening | 700 | 140 | EAPD |
| 2024 | Soares | 9 | BRazil | South America | Natal | hospital setting | 1021 | 114 | EAPD |
| 2024 | Etman | 6 | Egypt | Asia | Mansoura | hospital setting | 3000 | 375 | EAPD |
| 2024 | Lopes | 7 | Brazil | South America | Araçatuba | school screening | 500 | 116 | EAPD |
| 2024 | Lazo Huanqui | 9 | Peru | South America | Arequipa | school screening | 101 | 62 | EAPD |
| 2024 | Previero | 9 | Brazil | South America | São João da Boa Vista | school screening | 473 | 60 | EAPD |
| 2024 | Alkyildiz | 9 | Turkey | Asia | Aydın | school screening | 1010 | 53 | EAPD |
| 2024 | El Fitriyah | 7 | Indonesia | Asia | Cimahi City & West Bandung | school screening | 590 | 72 | EAPD |
| 2024 | Medina Varela | 9 | Mexico | South America | Ayala | school screening | 573 | 216 | EAPD |
| 2024 | Baghlaf | 9 | Saudi Arabia | Asia | Jeddah | school screening | 2010 | 359 | EAPD |
| 2024 | Susin | 7 | Brazil | South America | Porto Alegre | hospital setting | 82 | 28 | EAPD |
| 2024 | Ortega Luengo | 8 | Spain | Europe | Madrid | hospital setting | 489 | 140 | EAPD |
| 2024 | Zameer | 7 | Saudi Arabia | Asia | Jazan | hospital setting | 1405 | 112 | MIH-SSS (Cabral 2020) |
| 2024 | Osadolor | 9 | Nigeria | Africa | Nkanu-West & Udi | school screening | 296 | 7 | EAPD |

**Table S2.** Newcastle-Ottawa scale (NOS) for cross-sectional studies applied to selected MIH studies

*The Newcastle-Ottawa Scale (NOS) adapted for cross-sectional studies was used to assess study quality. Stars were assigned across selection, comparability, and outcome domains (maximum 9 stars). The total quality score was used to classify the risk of bias (RoB) as low (≥7), moderate (5–6), or high (≤4).*

| Author | **Selection:** (Maximum 5 stars) | | | | **Comparability:** (Maximum 2 stars) | **Outcome:** (Maximum 3 stars) | |  | |
| --- | --- | --- | --- | --- | --- | --- | --- | --- | --- |
|  | 1)Representativeness of the sample | 2) Sample size | 3) Non-respondents | 4) Ascertainment of the exposure (risk factor) | 5) The subjects in different outcome groups are comparable, based on the study design or analysis. Confounding factors are controlled. | 6) Assessment of the outcome | 7) Statistical test | Total quality score= | RoB Score |
| JaÈlevik 2001 | * | * | * | ** | * | ** | * | 9 | 9 (Low) |
| Dietrich 2003 | * | * |  | ** | * | * | * | 8 | 8 (Low) |
| Calderara 2005 | * | * | * | ** | * | ** | * | 9 | 9 (Low) |
| Fteita 2006 | * | * | * | ** | * | ** |  | 8 | 8 (Low) |
| Muratbegovic 2007 | * | * | * | * | * | ** | * | 8 | 8 (Low) |
| Jasulaitytò 2007 | * | * | * | ** | * | ** | * | 9 | 9 (Low) |
| Preusser 2007 | * | * | * | ** | * | ** | * | 9 | 9 (Low) |
| Cho 2008 | * | * | * | * | * | * | * | 7 | 7 (Low) |
| Kemoli 2008 | * | * | * | * | * | ** |  | 7 | 7 (Low) |
| Kukleva 2008 | * | * | * | * | * | ** |  | 7 | 7 (Low) |
| Kuscu 2008 |  |  | * | ** | * | ** | * | 7 | 7 (Low) |
| Lydiakis 2008 | * | * | * | ** | * | ** | * | 9 | 9 (Low) |
| Mahoney 2009 |  |  | * | ** | * | ** |  | 6 | 6 (Moderate) |
| Hasenauer 2010 | * | * | * | * | * | ** |  | 7 | 7 (Low) |
| Shin 2010 | * | * | * | * | * |  |  | 5 | 5 (Moderate) |
| Da Costa Silva 2010 | * | * | * | ** | * | ** | * | 9 | 9 (Low) |
| Zawaideh 2011 | * | * | * | ** | * | ** | * | 9 | 9 (Low) |
| Broga ̊rdh-Roth 2011 |  |  | * | ** | * | ** | * | 7 | 7 (Low) |
| Martinez Gomez 2011 |  |  | * | ** | * | ** | * | 7 | 7 (Low) |
| Jans 2011 |  |  | * | ** | * | ** |  | 6 | 6 (Moderate) |
| Ghanim 2011 | * | * | * | ** | * | ** | * | 9 | 9 (Low) |
| Ahmadi 2012 |  |  | * | ** | * | ** | * | 7 | 7 (Low) |
| Kühnisch 2012 | * | * | * | ** | * | ** | * | 9 | 9 (Low) |
| Elfrink 2012 | * | * | * | ** | * | ** | * | 9 | 9 (Low) |
| Păsăreanu 2012 |  |  | * | ** | * | ** | * | 7 | 7 (Low) |
| Parikh 2012 | * | * |  | ** | * | * | * | 8 | 8 (Low) |
| Ghanim 2012 | * | * | * | ** | * | ** | * | 9 | 9 (Low) |
| Heitmuller 2012 | * | * |  | ** | * | * | * | 8 | 8 (Low) |
| Ali Salih 2012 |  |  | * | ** | * | * | * | 6 | 6 (Moderate) |
| Biondi 2012 | * | * | * | ** | * | ** | * | 9 | 9 (Low) |
| Durmus 2013 |  |  |  | ** | * | ** | * | 6 | 6 (Moderate) |
| Sonmez 2013 | * | * | * | ** | * | ** | * | 9 | 9 (Low) |
| Jeremias 2013 | * | * | * | ** | * | ** | * | 9 | 9 (Low) |
| Grošelj 2013 |  |  | * | ** | * | ** | * | 7 | 7 (Low) |
| Noori 2014 | * | * | * | ** | * | ** | * | 9 | 9 (Low) |
| Jankovic 2014 |  |  | * | ** | * | ** | * | 7 | 7 (Low) |
| Pitiphat-A 2014 |  |  | * | ** | * | ** | * | 7 | 7 (Low) |
| Pitiphat-B 2014 |  |  | * | ** | * | ** | * | 7 | 7 (Low) |
| Woullett 2014 | * | * | * | ** | * | ** | * | 9 | 9 (Low) |
| Shrestha 2014 | * | * | * | ** | * | ** | * | 9 | 9 (Low) |
| Lopez Jordi 2014 | * | * | * | ** | * | ** | * | 9 | 9 (Low) |
| Petrou 2014 | * | * | * | ** | * | ** | * | 9 | 9 (Low) |
| Garcia-Margarit 2014 | * | * | * | ** | * | ** | * | 9 | 9 (Low) |
| Ng 2014 | * | * | * | ** | * | ** | * | 9 | 9 (Low) |
| Pruneda 2014 |  |  | * | ** | * | ** | * | 7 | 7 (Low) |
| Allazzam 2014 |  |  | * | ** | * | ** | * | 7 | 7 (Low) |
| Bhaskar 2014 | * | * | * | ** | * | ** | * | 9 | 9 (Low) |
| Haidary 2014 | * | * | * | ** | * | ** | * | 9 | 9 (Low) |
| Mittal 2014 | * | * | * | ** | * | ** | * | 9 | 9 (Low) |
| Temilola 2015 | * | * | * | ** | * | ** | * | 9 | 9 (Low) |
| Lima 2015 |  |  | * | ** | * | ** |  | 6 | 6 (Moderate) |
| Balmer 2015 | * | * | * | ** | * | ** | * | 9 | 9 (Low) |
| Oyedele 2015 |  |  | * | ** | * | ** |  | 6 | 6 (Moderate) |
| Krishnan 2015 | * | * | * | ** | * | ** |  | 8 | 8 (Low) |
| Ofi 2015 |  |  | * | ** | * | ** | * | 7 | 7 (Low) |
| Kirthiga 2015 | * | * | * | ** | * | ** | * | 9 | 9 (Low) |
| Kairala 2015 |  |  | * | ** | * | ** | * | 7 | 7 (Low) |
| Hanan 2015 | * | * | * | ** | * | ** | * | 9 | 9 (Low) |
| Kevrekidou 2015 | * | * | * | ** | * | ** | * | 9 | 9 (Low) |
| Tadikonda 2015 |  |  | * | ** | * | ** | * | 7 | 7 (Low) |
| Hussein 2015 |  |  | * | ** | * | ** |  | 6 | 6 (Moderate) |
| Yannam 2016 | * | * | * | ** | * | ** |  | 8 | 8 (Low) |
| Tourino 2016 | * | * | * | ** | * | ** | * | 9 | 9 (Low) |
| Hussain 2016 |  |  | * | ** | * | ** | * | 7 | 7 (Low) |
| Garot 2016 | * | * | * | ** | * | ** | * | 9 | 9 (Low) |
| Siddaiah 2016 | * | * | * | ** | * | ** | * | 9 | 9 (Low) |
| Ullah 2016 |  |  | * | ** | * | ** | * | 7 | 7 (Low) |
| Schmalfuss 2016 | * | * | * | ** | * | ** | * | 9 | 9 (Low) |
| Negre-Barber 2016 |  |  | * | ** | * | ** | * | 7 | 7 (Low) |
| Martinovic 2016 | * | * | * | ** | * | ** | * | 9 | 9 (Low) |
| Andrade 2016 |  |  | * | ** | * | ** | * | 7 | 7 (Low) |
| Mishra 2016 | * | * | * | ** | * | ** | * | 9 | 9 (Low) |
| Kim 2016 | * | * | * | ** | * | ** | * | 9 | 9 (Low) |
| Hysi 2016 | * | * | * | ** | * | ** |  | 8 | 8 (Low) |
| Quintana 2017 |  |  | * | ** | * | ** | * | 7 | 7 (Low) |
| Hong 2017 | * | * | * | ** | * | ** | * | 9 | 9 (Low) |
| Gurrusquieta 2017 | * | * | * | ** | * | ** | * | 9 | 9 (Low) |
| Almaz 2017 | * | * | * | ** | * | ** |  | 8 | 8 (Low) |
| Figueiredo 2017 | * | * | * | ** | * | ** | * | 9 | 9 (Low) |
| Sidaly 2017 |  |  | * | ** | * | ** | * | 7 | 7 (Low) |
| Shin 2017 | * | * | * | ** | * | ** | * | 9 | 9 (Low) |
| Poureslami 2017 | * | * | * | ** | * | ** | * | 9 | 9 (Low) |
| Priya 2017 | * | * | * | ** | * | ** | * | 9 | 9 (Low) |
| Buchraber 2017 | * | * | * | ** | * | ** | * | 9 | 9 (Low) |
| Lago 2017 |  |  | * | ** | * | ** | * | 7 | 7 (Low) |
| Baakkal 2017 |  |  | * | ** | * | ** | * | 7 | 7 (Low) |
| Mulic 2017 |  |  | * | ** | * | ** | * | 7 | 7 (Low) |
| Koruyucu 2018 | * | * | * | ** | * | ** | * | 9 | 9 (Low) |
| Hernández 2018 | * | * | * | ** | * | ** |  | 8 | 8 (Low) |
| Folayan-A 2018 | * | * | * | ** | * | ** | * | 9 | 9 (Low) |
| Folayan-~~B~~ 2018 | * | * | * | ** | * | ** | * | 9 | 9 (Low) |
| Saitoh 2018 | * | * | * | ** | * | ** | * | 9 | 9 (Low) |
| Teixeira 2018 |  |  | * | ** | * | ** | * | 7 | 7 (Low) |
| Gambetta-Tessini 2018 |  |  | * | ** | * | ** | * | 7 | 7 (Low) |
| Saber 2018 | * | * | * | ** | * | ** |  | 8 | 8 (Low) |
| Rai 2018 | * | * | * | ** | * | ** | * | 9 | 9 (Low) |
| Samuel 2018 | * | * | * | ** | * | ** | * | 9 | 9 (Low) |
| Padavala 2018 |  |  | * | ** | * | ** |  | 6 | 6 (Moderate) |
| Dantas-Neta 2018 |  |  | * | ** | * | ** | * | 7 | 7 (Low) |
| Al-Hammad 2018 | * | * | * | ** | * | ** | * | 9 | 9 (Low) |
| Ordonez-Romero 2019 |  |  | * | ** | * | ** | * | 7 | 7 (Low) |
| Medina 2019 |  |  | * | ** | * | ** | * | 7 | 7 (Low) |
| Maldonado 2019 |  |  | * | ** | * | ** | * | 7 | 7 (Low) |
| Irigoyen-Camacho 2019 |  |  | * | ** | * | ** | * | 7 | 7 (Low) |
| Praptiwi 2019 | * | * | * | ** | * | ** |  | 8 | 8 (Low) |
| Ardini 2019 |  |  | * | ** | * | ** |  | 6 | 6 (Moderate) |
| Menoncin 2019 | * | * | * | ** | * | ** | * | 9 | 9 (Low) |
| Glodkowska 2019 | * | * | * | ** | * | ** | * | 9 | 9 (Low) |
| Santos 2019 | * | * | * | ** | * | ** | * | 9 | 9 (Low) |
| Rai 2019 | * | * | * | ** | * | ** | * | 9 | 9 (Low) |
| Kılınç 2019 | * | * | * | ** | * | ** | * | 9 | 9 (Low) |
| Herojit Singh 2019 | * | * | * | ** | * | ** | * | 9 | 9 (Low) |
| Portella 2019 | * | * | * | ** | * | ** | * | 9 | 9 (Low) |
| Reyes 2019 | * | * | * | ** | * | ** | * | 9 | 9 (Low) |
| Goswami 2019 | * | * | * | ** | * | ** |  | 8 | 8 (Low) |
| Mejia 2019 | * | * | * | ** | * | ** | * | 9 | 9 (Low) |
| Davenport 2019 |  |  | * | ** | * | ** | * | 7 | 7 (Low) |
| Agarwal 2019 | * | * | * | ** | * | ** | * | 9 | 9 (Low) |
| Villanueva Gutiérrez 2019 |  |  | * | ** | * | ** | * | 7 | 7 (Low) |
| Glodkowska 2020 | * | * | * | ** | * | ** | * | 9 | 9 (Low) |
| Einollahi 2020 |  |  | * | ** | * | ** | * | 7 | 7 (Low) |
| Da Silva 2020 |  |  | * | ** | * | ** | * | 7 | 7 (Low) |
| Zafar 2020 |  |  | * | ** | * | ** |  | 6 | 6 (Moderate) |
| Fernandes 2020 |  |  | * | ** | * | ** | * | 7 | 7 (Low) |
| Hamdan 2020 | * | * | * | ** | * | ** | * | 9 | 9 (Low) |
| Sakly 2020 | * | * | * | ** | * | ** |  | 8 | 8 (Low) |
| Jurlina 2020 | * | * | * | ** | * | ** |  | 8 | 8 (Low) |
| Wogelius 2020 | * | * | * | ** | * | ** | * | 9 | 9 (Low) |
| Sidhu 2020 |  |  | * | ** | * | ** | * | 7 | 7 (Low) |
| Fragelli 2020 | * | * | * | ** | * | ** | * | 9 | 9 (Low) |
| Tagelsir Ahmed 2020 |  |  | * | ** | * | ** | * | 7 | 7 (Low) |
| Bahrololoomi 2020 |  |  | * | ** | * | ** | * | 7 | 7 (Low) |
| Elzein 2020 |  |  | * | ** | * | ** |  | 6 | 6 (Moderate) |
| Singh 2020 |  |  | * | ** | * | ** |  | 6 | 6 (Moderate) |
| Ray 2020 | * | * | * | ** | * | ** | * | 9 | 9 (Low) |
| Emmatty 2020 | * | * | * | ** | * | ** | * | 9 | 9 (Low) |
| Ganapathi 2020 | * | * | * | ** | * | ** |  | 8 | 8 (Low) |
| Bulani 2020 | * | * | * | ** | * | ** | * | 9 | 9 (Low) |
| Hoyte 2020 |  |  | * | ** | * | ** | * | 7 | 7 (Low) |
| Tseveenjav 2020 | * | * | * | ** | * | ** | * | 9 | 9 (Low) |
| Gorni Dos Reis 2020 |  |  | * | ** | * | ** | * | 7 | 7 (Low) |
| Shojaeepour 2020 | * | * | * | ** | * | ** | * | 9 | 9 (Low) |
| Dourado 2021 |  |  | * | ** | * | ** | * | 7 | 7 (Low) |
| Manzoor 2021 |  |  | * | ** | * | ** |  | 6 | 6 (Moderate) |
| Alhowaish 2021 | * | * | * | ** | * | ** | * | 9 | 9 (Low) |
| Bonzanini 2021 |  |  | * | ** | * | ** | * | 7 | 7 (Low) |
| Arheiam 2021 | * | * | * | ** | * | ** | * | 9 | 9 (Low) |
| Almuallem 2021 | * | * | * | ** | * | ** |  | 8 | 8 (Low) |
| Rodriguez 2021 |  |  | * | ** | * | ** | * | 7 | 7 (Low) |
| Padmanabhan 2021 | * | * | * | ** | * | ** | * | 9 | 9 (Low) |
| Khazaei 2021 | * | * | * | ** | * | ** | * | 9 | 9 (Low) |
| Rajic 2021 |  |  | * | ** | * | ** | * | 7 | 7 (Low) |
| Ravichandra 2021 | * | * | * | ** | * | ** | * | 9 | 9 (Low) |
| Hali 2021 | * | * | * | ** | * | ** |  | 8 | 8 (Low) |
| Abdalla 2021 |  |  | * | ** | * | ** | * | 7 | 7 (Low) |
| Bibin 2021 |  |  | * | ** | * | ** |  | 6 | 6 (Moderate) |
| Chavez-Cruz 2021 |  |  | * | ** | * | ** | * | 7 | 7 (Low) |
| Freitas Fernandes 2021 |  |  | * | ** | * | ** | * | 7 | 7 (Low) |
| Farias 2021 |  |  | * | ** | * | ** | * | 7 | 7 (Low) |
| Mohamed 2021 |  |  | * | ** | * | ** | * | 7 | 7 (Low) |
| Abdelaziz 2022 | * | * | * | ** | * | ** |  | 8 | 8 (Low) |
| Mariam 2022 | * | * | * | ** | * | ** | * | 9 | 9 (Low) |
| Khan 2022 | * | * | * | ** | * | ** | * | 9 | 9 (Low) |
| El Ghaffar 2022 |  |  | * | ** | * | ** | * | 7 | 7 (Low) |
| Zhang 2022 | * | * | * | ** | * | ** | * | 9 | 9 (Low) |
| Børsting 2022 |  |  | * | ** | * | ** | * | 7 | 7 (Low) |
| Vanhée 2022 |  |  | * | ** | * | ** | * | 7 | 7 (Low) |
| Verma 2022 | * | * | * | ** | * | ** | * | 9 | 9 (Low) |
| Argote Quispe 2022 |  |  | * | ** | * | ** | * | 7 | 7 (Low) |
| Nisii 2022 | * | * | * | ** | * | ** | * | 9 | 9 (Low) |
| Quintero 2022 |  |  | * | ** | * | * | * | 6 | 6 (Moderate) |
| Grieshaber 2022 | * | * | * | ** | * | ** | * | 9 | 9 (Low) |
| Ilczuk-Rypuła 2022 | * | * | * | ** | * | ** | * | 9 | 9 (Low) |
| Ciocan 2023 |  |  | * | ** | * | * | * | 6 | 6 (Moderate) |
| Al‐Nerabieah 2023 | * | * | * | ** | * | ** | * | 9 | 9 (Low) |
| Cots 2024 | * | * | * | ** | * | ** |  | 8 | 8 (Low) |
| Tessari 2024 | * | * | * | ** | * | ** | * | 9 | 9 (Low) |
| Chowdhury 2024 | * | * | * | ** | * | ** |  | 8 | 8 (Low) |
| Afzal 2024 | * | * | * | ** | * | ** |  | 8 | 8 (Low) |
| Freitas Fernandes 2024 | * | * | * | ** | * | ** | * | 9 | 9 (Low) |
| Sharma 2024 | * | * | * | ** | * | ** | * | 9 | 9 (Low) |
| Al Jeghami 2024 |  |  | * | ** | * | ** | * | 7 | 7 (Low) |
| Brejawi 2024 |  |  | * | ** | * | ** | * | 7 | 7 (Low) |
| Seloglu 2024 |  |  | * | ** | * | ** | * | 7 | 7 (Low) |
| Abdulla 2024 |  |  | * | ** | * | ** | * | 7 | 7 (Low) |
| Soares 2024 | * | * | * | ** | * | ** | * | 9 | 9 (Low) |
| Etman 2024 |  |  | * | ** | * | ** |  | 6 | 6 (Moderate) |
| Lopes 2024 |  |  | * | ** | * | ** | * | 7 | 7 (Low) |
| Lazo Huanqui 2024 | * | * | * | ** | * | ** | * | 9 | 9 (Low) |
| Previero 2024 | * | * | * | ** | * | ** | * | 9 | 9 (Low) |
| Alkyildiz 2024 | * | * | * | ** | * | ** | * | 9 | 9 (Low) |
| El Fitriyah 2024 |  |  | * | ** | * | ** | * | 7 | 7 (Low) |
| Medina Varela 2024 | * | * | * | ** | * | ** | * | 9 | 9 (Low) |
| Baghlaf 2024 | * | * | * | ** | * | ** | * | 9 | 9 (Low) |
| Susin 2024 |  |  | * | ** | * | ** | * | 7 | 7 (Low) |
| Ortega Luengo 2024 | * | * | * | ** | * | ** |  | 8 | 8 (Low) |
| Zameer 2024 |  |  | * | ** | * | ** | * | 7 | 7 (Low) |
| Osadolor 2024 | * | * | * | ** | * | ** | * | 9 | 9 (Low) |

**Table S3**. Main characteristics of diagnostic indices used in epidemiological studies on MIH

*MIH-specific’ indicates whether the index was specifically developed for molar incisor hypomineralization (MIH). The term ‘partly’ refers to indices that were not originally designed for MIH but include features applicable to MIH diagnosis. ‘Severity component’ refers to the presence and extent of severity grading within the index; ‘limited’ indicates basic or partial severity categorization, while ‘variable’ reflects differences in severity definitions and thresholds across studies using the same index.*

.

| **Diagnostic index** | **Main scope** | **Key diagnostic features** | **MIH-specific** | **Severity component** | **Main limitation for epidemiological comparison** |
| --- | --- | --- | --- | --- | --- |
| EAPD | Epidemiological and clinical diagnosis of MIH | Demarcated opacities, post-eruptive breakdown, atypical restorations, extracted molars due to MIH | Yes | Limited | Examiner-dependent interpretation of mild lesions |
| mDDE | General developmental enamel defects | Records enamel defects broadly, not specific for MIH | No | Limited | Risk of including non-MIH defects |
| Mathu-Maju and Wright | Clinical description of MIH | Focus on characteristic clinical presentation and management-oriented description | Partly | Limited | Less widely adopted internationally |
| Wetzel and Reckel | Early description of molar defects | Earlier classification of defective molars | No / historical | No | Limited standardization and current use |
| FDI | General enamel defect assessment | Broader enamel defect framework | No | Variable | May capture wider spectrum of lesions |
| MIH-SSS | Severity-based MIH assessment | Structured scoring according to lesion severity | Yes | Yes | Greater complexity for field studies |
| MIH-TNI | Treatment need and severity | Clinical severity and treatment-oriented categorization | Yes | Yes | More suitable for clinical than epidemiological use |

**References in the Supplementary:**

1. Jälevik B, Norén JG, Klingberg G, Barregård L. (2001) Etiologic factors influencing the prevalence of demarcated opacities in permanent first molars in a group of Swedish children. ;109:230-234 doi:10.1034/j.1600-0722.2001.00047.x

2. Dietrich G, Sperling S, Hetzer G. (2003) Molar incisor hypomineralisation in a group of children and adolescents living in Dresden (Germany). Eur J Paediatr Dent. Sep ;4(3):133-7.

3. Calderara PC, Gerthoux PM, Mocarelli P, Lukinmaa PL, Tramacere PL, Alaluusua S. (2005) The prevalence of Molar Incisor Hypomineralisation (MIH) in a group of Italian school children. Eur J Paediatr Dent. Jun ;6(2):79-83.

4. Fteita D, Ali A, Alaluusua S. (2006) Molar-incisor hypomineralization (MIH) in a group of school-aged children in Benghazi, Libya. Eur Arch Paediatr Dent. Jun ;7(2):92-5. doi:10.1007/BF03320821

5. Muratbegovic A, Markovic N, Ganibegovic Selimovic M. (2007) Molar incisor hypomineralisation in Bosnia and Herzegovina: aetiology and clinical consequences in medium caries activity population. Eur Arch Paediatr Dent. Dec ;8(4):189-94. doi:10.1007/BF03262595

6. Jasulaityte L, Veerkamp JS, Weerheijm KL. (2007) Molar incisor hypomineralization: review and prevalence data from the study of primary school children in Kaunas/Lithuania. Eur Arch Paediatr Dent. Jun ;8(2):87-94. doi:10.1007/BF03262575

7. Preusser SE, Ferring V, Wleklinski C, Wetzel WE. (2007) Prevalence and severity of molar incisor hypomineralization in a region of Germany -- a brief communication. J Public Health Dent. ;67(3):148-50. doi:10.1111/j.1752-7325.2007.00040.x

8. Cho SY, Ki Y, Chu V. (2008) Molar incisor hypomineralization in Hong Kong Chinese children. Int J Paediatr Dent. Sep ;18(5):348-52. doi:10.1111/j.1365-263X.2008.00927.x

9. Kemoli AM. (2008) Prevalence of molar incisor hypomineralisation in six to eight year-olds in two rural divisions in Kenya. East Afr Med J. Oct ;85(10):514-9. doi:10.4314/eamj.v85i10.9668

10. Kukleva MP, Petrova SG, Kondeva VK, Nihtyanova TI. (2008) Molar incisor hypomineralisation in 7-to-14-year old children in Plovdiv, Bulgaria--an epidemiologic study. Folia Med (Plovdiv). ;50(3):71-5.

11. Kuscu OO, Caglar E, Aslan S, Durmusoglu E, Karademir A, Sandalli N. (2009) The prevalence of molar incisor hypomineralization (MIH) in a group of children in a highly polluted urban region and a windfarm-green energy island. Int J Paediatr Dent. May ;19(3):176-85. doi:10.1111/j.1365-263X.2008.00945.x

12. Lygidakis NA, Dimou G, Briseniou E. (2008) Molar-incisor-hypomineralisation (MIH). Retrospective clinical study in Greek children. I. Prevalence and defect characteristics. Eur Arch Paediatr Dent. ;9(4):200-6. doi:10.1007/BF03262636

13. Mahoney EK, Morrison DG. (2009) The prevalence of Molar-Incisor Hypomineralisation (MIH) in Wainuiomata children. N Z Dent J. Dec ;105(4):121-7.

14. Hasenauer L, Vogelsberger M, Bürkle V, Grunert I, Meißner N. (2010) Prävalenz und Ausprägung der Molar Incisor Hypomineralisation (MIH) in Salzburg und Tirol und ein Beitrag zur Erforschung der Ursachen. . Stomatologie. ;107:43-50. doi:10.1007/s00715-010-0118-5

15. Shin JH, An UJ, Kim S, Jeong TS. (2010) The prevalence of molar incisor hypomineralization and status of first molars in primary school children. JKAPD. ;37(2):179-85.

16. da Costa-Silva CM, Jeremias F, de Souza JF, Cordeiro ReC, Santos-Pinto L, Zuanon AC. (2010) Molar incisor hypomineralization: prevalence, severity and clinical consequences in Brazilian children. Int J Paediatr Dent. Nov ;20(6):426-34. doi:10.1111/j.1365-263X.2010.01097.x

17. Zawaideh FI, Al-Jundi SH, Al-Jaljoli MH. (2011) Molar incisor hypomineralisation: prevalence in Jordanian children and clinical characteristics. Eur Arch Paediatr Dent. Feb ;12(1):31-6. doi:10.1007/BF03262776

18. Brogårdh-Roth S, Matsson L, Klingberg G. (2011) Molar-incisor hypomineralization and oral hygiene in 10- to-12-yr-old Swedish children born preterm. Eur J Oral Sci. Feb ;119(1):33-9. doi:10.1111/j.1600-0722.2011.00792.x

19. Martinez Gomez TP, Guinot Jimeno F, Bellet Dalmau LJ, Giner Tarrida L. (2012) Prevalence of molar–incisor hypomineralisation observed using transillumination in a group of children from Barcelona (Spain). International Journal of Paediatric Dentistry. ;22(2):100-109.

20. Jans MA, Diaz MJ, Vergara GC, Zaror SC. (2011) Frequency and severity of the molar incisor hypomineralization in patients treated at the dental clinic of the Universidad de La Frontera, Chile. Int J Odontostomatol. ;5(2):133-140.

21. Ghanim A, Morgan M, Mariño R, Bailey D, Manton D. (2011) Molar-incisor hypomineralisation: prevalence and defect characteristics in Iraqi children. Int J Paediatr Dent. Nov ;21(6):413-21. doi:10.1111/j.1365-263X.2011.01143.x

22. Ahmadi R, Ramazani N, Nourinasab R. (2012) Molar incisor hypomineralization: a study of prevalence and etiology in a group of Iranian children. Iran J Pediatr. Jun ;22(2):245-51.

23. Kühnisch J, Heitmüller D, Thiering E, et al. (2014) Proportion and extent of manifestation of molar-incisor-hypomineralizations according to different phenotypes. J Public Health Dent. ;74(1):42-9. doi:10.1111/j.1752-7325.2012.00365.x

24. Elfrink ME, ten Cate JM, Jaddoe VW, Hofman A, Moll HA, Veerkamp JS. (2012) Deciduous molar hypomineralization and molar incisor hypomineralization. J Dent Res. Jun ;91(6):551-5. doi:10.1177/0022034512440450

25. Păsăreanu M, Mocanu RM, Bălan A. (2012) The syndrome MIH systemic impact in children and adolescents: relevance area. Romanian Journal of Oral Rehabilitation. ;4(2):82-89.

26. Parikh DR, Ganesh M, Bhaskar V. (2012) Prevalence and characteristics of Molar Incisor Hypomineralisation (MIH) in the child population residing in Gandhinagar, Gujarat, India. Eur Arch Paediatr Dent. Feb ;13(1):21-6. doi:10.1007/BF03262836

27. Ghanim A, Bagheri R, Golkari A, Manton D. (2014) Molar–incisor hypomineralisation: a prevalence study amongst primary schoolchildren of Shiraz, Iran. European Archives of Paediatric Dentistry. ;15:75-82. doi:10.1007/s40368-013-0067-y

28. Heitmüller D, Thiering E, Hoffmann U, et al. (2013) Is there a positive relationship between molar incisor hypomineralisations and the presence of dental caries? Int J Paediatr Dent. Mar ;23(2):116-24. doi:10.1111/j.1365-263X.2012.01233.x

29. Salih BA, Khalaf MS. (2012) Prevalence of molar-incisorhypomineralization among children attending pedodontic clinic of college of dentistry at Baghdad University. J Bagh Coll Dentistry. ;24(4):121-125.

30. Biondi AM, López Jordi MDC, Cortese SG, Álvarez L, Salveraglio I, Ortolani AM. (2012) Prevalence of molar-incisor hypomineralization (MIH) in children seeking dental care at the Schools of Dentistry of the University of Buenos Aires (Argentina) and University of la Republica (Uruguay). Acta Odontológica Latinoamericana. ;25(2):224-230.

31. Durmus B, Abbasoglu Z, Peker S, Kargul B. (2013) Possible medical aetiological factors and characteristics of molar incisor hypomineralisation in a group of Turkish children/Moguci medicinski etioloski cimbenici i znacajke molarno incizivne hipomineralizacije u skupini turske djece. Acta Stomatologica Croatica. ;47(4):297-306.

32. Sönmez H, Yıldırım G, Bezgin T. (2013) Putative factors associated with molar incisor hypomineralisation: an epidemiological study. Eur Arch Paediatr Dent. ;14(6):375-380. doi:10.1007/s40368-013-0012-0

33. Jeremias F, Souza JFD, Costa Silva CMD, Cordeiro RDCL, Zuanon ÂCC, Santos-Pinto L. (2013) Dental caries experience and molar-incisor hypomineralization. Acta Odontologica Scandinavica. ;71(3-4):870-876.

34. Grošelj M, Jan J. (2013) Molar incisor hypomineralisation and dental caries among children in Slovenia. Eur J Paediatr Dent. Sep ;14(3):241-5.

35. Noori A, Hussein S. (2014) Molar-incisor hypomineralisation (MIH) among Kurdish children in Sulaimani City, Iraq. Sulaimani Dental Journal. ;1:45-50. doi:10.17656/sdj.10019

36. Janković S, Ivanović M, Davidović B, Lecić J. (2014) Distribution and characteristics of molar-incisor hypomineralization. Vojnosanit Pregl. Aug ;71(8):730-4. doi:10.2298/vsp1408730j

37. Pitiphat W, Savisit R, Chansamak N, Subarnbhesaj A. (2014) Molar incisor hypomineralization and dental caries in six- to seven-year-old Thai children. Pediatr Dent. ;36(7):478-82.

38. Pitiphat W, Luangchaichaweng S, Pungchanchaikul P, Angwaravong O, Chansamak N. (2014) Factors associated with molar incisor hypomineralization in Thai children. Eur J Oral Sci. Aug ;122(4):265-70. doi:10.1111/eos.12136

39. Wuollet E, Laisi S, Salmela E, Ess A, Alaluusua S. (2014) Background factors of molar-incisor hypomineralization in a group of Finnish children. Acta Odontol Scand. Nov ;72(8):963-9. doi:10.3109/00016357.2014.931459

40. Shrestha R, Upadhaya S, Bajracharya M. (2014) Prevalence of molar incisor hypomineralisation among school children in Kavre. Kathmandu Univ Med J (KUMJ). ;12(45):38-42. doi:10.3126/kumj.v12i1.13631

41. López Jordi Mdel C, Cortese SG, Álvarez L, Salveraglio I, Ortolani AM, Biondi AM. (2014) Comparison of the prevalence of molar incisor hypomineralization among children with different health care coverage in the cities of Buenos Aires (Argentina) and Montevideo (Uruguay). Salud Colect. ;10(2)doi:10.1590/S1851-82652014000200008

42. Petrou MA, Giraki M, Bissar AR, et al. (2014) Prevalence of Molar-Incisor-Hypomineralisation among school children in four German cities. Int J Paediatr Dent. Nov ;24(6):434-40. doi:10.1111/ipd.12089

43. Garcia-Margarit M, Catalá-Pizarro M, Montiel-Company JM, Almerich-Silla JM. (2014) Epidemiologic study of molar-incisor hypomineralization in 8-year-old Spanish children. Int J Paediatr Dent. Jan ;24(1):14-22. doi:10.1111/ipd.12020

44. Ng JJ, Eu OC, Nair R, Hong CH. (2015) Prevalence of molar incisor hypomineralization (MIH) in Singaporean children. Int J Paediatr Dent. Mar ;25(2):73-8. doi:10.1111/ipd.12100

45. Murrieta-Pruneda JF, Torres-Vargas J. (2014) Frequency and Severity of Molar Incisor HypoMineralization (HIM) in a group of Mexican children. Frequency and Severity of Molar Incisor HypoMineralization (HIM) in a group of Mexican children. ;12(23):7-14.

46. Allazzam SM, Alaki SM, El Meligy OA. (2014) Molar incisor hypomineralization, prevalence, and etiology. Int J Dent. ;2014:234508. doi:10.1155/2014/234508

47. Bhaskar SA, Hegde S. (2014) Molar-incisor hypomineralization: prevalence, severity and clinical characteristics in 8- to 13-year-old children of Udaipur, India. J Indian Soc Pedod Prev Dent. ;32(4):322-9. doi:10.4103/0970-4388.140960

48. Haidary S. (2015) Comparison of the Prevalence of Molar Incisor Hypomineralization in Dubai/United Arab Emirate and Greifswald/Germany. .

49. Mittal NP, Goyal A, Gauba K, Kapur A. (2014) Molar incisor hypomineralisation: prevalence and clinical presentation in school children of the northern region of India. Eur Arch Paediatr Dent. Feb ;15(1):11-8. doi:10.1007/s40368-013-0045-4

50. Temilola OD, Folayan MO. (2015) Distinguishing predisposing factors for enamel hypoplasia and molar-incisor hypomineralization in children in Ile-Ife, Nigeria. Brazilian Journal of Oral Sciences. ;14:318-322

51. de Lima MeD, Andrade MJ, Dantas-Neta NB, et al. (2015) Epidemiologic Study of Molar-incisor Hypomineralization in Schoolchildren in North-eastern Brazil. Pediatr Dent. ;37(7):513-9.

52. Balmer R, Toumba KJ, Munyombwe T, Godson J, Duggal MS. (2015) The prevalence of incisor hypomineralisation and its relationship with the prevalence of molar incisor hypomineralisation. Eur Arch Paediatr Dent. Jun ;16(3):265-9. doi:10.1007/s40368-014-0171-7

53. Oyedele TA, Folayan MO, Adekoya-Sofowora CA, Oziegbe EO, Esan TA. (2015) Prevalence, pattern and severity of molar incisor hypomineralisation in 8- to 10-year-old school children in Ile-Ife, Nigeria. Eur Arch Paediatr Dent. Jun ;16(3):277-82. doi:10.1007/s40368-015-0175-y

54. Krishnan R, Ramesh M, Chalakkal P. (2015) Prevalence and characteristics of MIH in school children residing in an endemic fluorosis area of India: an epidemiological study. Eur Arch Paediatr Dent. ;16(6):455-460. doi:10.1007/s40368-015-0194-8

55. Ofi WA, Salih BA. (2015) Prevalence and severity of molar-incisor hypomineralisation with relation to its etiological factors among school children 7-9 years of Al-Najaf governorate. Journal of Baghdad College of Dentistry. ;27(3):169-173.

56. Kirthiga M, Poornima P, Praveen R, Gayathri P, Manju M, Priya M. (2015) Prevalence and severity of molar incisor hypomineralization in children aged 11-16 years of a city in Karnataka, Davangere. J Indian Soc Pedod Prev Dent. ;33(3):213-7. doi:10.4103/0970-4388.160366

57. Kairala ALR. (2015) Hipomineralização molar incisivo (MIH): uma correlação de fatores possivelmente desencadeantes. UNIVERSIDADE DE BRASÍLIA;

58. Hanan SA, de Oliveira Alves Filho A, Medina PO, Cordeiro RDCL, Santos-Pinto L, Zuanon ÂCC. (2015) Molar-incisor hypomineralization in schoolchildren of Manaus, Brazil. Pesquisa Brasileira em Odontopediatria e Clínica Integrada. ;15(1):309-317.

59. Kevrekidou A, Kosma I, Arapostathis K, Kotsanos N. (2015) Molar Incisor Hypomineralization of Eight- and 14-year-old Children: Prevalence, Severity, and Defect Characteristics. Pediatr Dent. ;37(5):455-461.

60. Tadikonda AN, Acharya S, Pentapati KC. (2015) Prevalence of molar incisor hypomineralization and its relation with dental caries in school children of Udupi district, South India. World Journal of Dentistry. ;6(3):143-146.

61. Hussein AS, Faisal M, Haron M, Ghanim AM, Abu-Hassan MI. (2015) Distribution of Molar Incisor Hypomineralization in Malaysian Children Attending University Dental Clinic. J Clin Pediatr Dent. ;39(3):219-23. doi:10.17796/1053-4628-39.3.219

62. Yannam SD, Amarlal D, Rekha CV. (2016) Prevalence of molar incisor hypomineralization in school children aged 8-12 years in Chennai. J Indian Soc Pedod Prev Dent. ;34(2):134-8. doi:10.4103/0970-4388.180438

63. Tourino LF, Corrêa-Faria P, Ferreira RC, Bendo CB, Zarzar PM, Vale MP. (2016) Association between Molar Incisor Hypomineralization in Schoolchildren and Both Prenatal and Postnatal Factors: A Population-Based Study. PLoS One. ;11(6):e0156332. doi:10.1371/journal.pone.0156332

64. Hussain G. The prevalence and severity of molar incisor hypomineralisation in Dubai, uae, a cross sectional study. (2016) Mohammed Bin Rashid University of Medicine and Health Sciences; .

65. Garot E, Manton D, Rouas P. (2016) Peripartum events and molar-incisor hypomineralisation (MIH) amongst young patients in southwest France. Eur Arch Paediatr Dent. Aug ;17(4):245-50. doi:10.1007/s40368-016-0235-y

66. Siddaiah SB, Thimmegowda U, Parameshwara PM, Ramachandra JA. (2016) Molar Incisor Hypomineralization: a study of prevalence and etiology in a group of south Bangalore children. International Journal of Current Research. ;8(12):43784-43788.

67. Ullah I, Naghma P, Raheela S. (2016) Pattern and Presentation of Molar Incisor Hypomineralizaion in Pakistani Children. Int J Contemp Med Res. ;3:724-726.

68. Schmalfuss A, Stenhagen KR, Tveit AB, Crossner CG, Espelid I. (2016) Canines are affected in 16-year-olds with molar–incisor hypomineralisation (MIH): an epidemiological study based on the Tromsø study:" Fit Futures". European Archives of Paediatric Dentistry. ;17(2):107-113.

69. Negre-Barber A, Montiel-Company JM, Boronat-Catalá M, Catalá-Pizarro M, Almerich-Silla JM. (2016) Hypomineralized Second Primary Molars as Predictor of Molar Incisor Hypomineralization. Sci Rep. Aug 25 ;6:31929. doi:10.1038/srep31929

70. Martinović B, Ivanović M, Cvetković A, et al. (2017) Prevalence, characteristics and severity of hypomineralization of the first permanent molars and incisors in children from the northern part of Kosovo and Metohija. Srpski arhiv za celokupno lekarstvo. ;145(7-8):364-69.

71. Andrade NS, Pontes AS, Paz HES, de Moura MS, Moura LF, Lima MD. (2017) Molar incisor hypomineralization in HIV-infected children and adolescents. Spec Care Dentist. Jan ;37(1):28-37. doi:10.1111/scd.12209

72. Mishra A, Pandey RK. (2016) Molar Incisor Hypomineralization: An Epidemiological Study with Prevalence and Etiological Factors in Indian Pediatric Population. Int J Clin Pediatr Dent. ;9(2):167-71. doi:10.5005/jp-journals-10005-1357

73. Kim T, Jeong I, Lee D, Kim J, Yang Y. (2016) Prevalence and etiology of molar incisor hypomineralization in children aged 8-9 years. Journal of the Korean Academy of Pediatric Dentistry. ;43(4):410-418.

74. Hysi D, Kuscu OO, Droboniku E, Toti C, Xhemnica L, Caglar E. (2016) Prevalence and aetiology of Molar-Incisor Hypomineralisation among children aged 8-10 years in Tirana, Albania. Eur J Paediatr Dent. Mar ;17(1):75-9.

75. Quintana Guachamín MA. (2017) Prevalencia de hipomineralización incisivo-molar en niños de 6 a 9 años de edad. .

76. Hong YL, Wu H, Yuan J, Yao H, Sui W, et al. (2017) . Prevalence Study of Molar-Incisor Hypo Mineralisation in Primary School Children in South China. J Pediatr Care. 2017;3(1):1-8.

77. Gurrusquieta BJ, Núñez VM, López ML. (2017) Prevalence of Molar Incisor Hypomineralization in Mexican Children. J Clin Pediatr Dent. ;41(1):18-21. doi:10.17796/1053-4628-41.1.18

78. Almaz ME, Sönmez IS, Oba AA. (2017) Prevalence and distribution of developmental dental anomalies in pediatric patients. Meandros Medical and Dental Journal. ;18(12):130.

79. da Silva Figueiredo Sé MJ, Ribeiro APD, Dos Santos-Pinto LAM, de Cassia Loiola Cordeiro R, Cabral RN, Leal SC. (2017) Are Hypomineralized Primary Molars and Canines Associated with Molar-Incisor Hypomineralization? Pediatr Dent. Nov 01 ;39(7):445-449.

80. Sidaly R, Schmalfuss A, Skaare AB, Sehic A, Stiris T, Espelid I. (2016) Five-minute Apgar score ≤ 5 and Molar Incisor Hypomineralisation (MIH) - a case control study. BMC Oral Health. ;17(1):25. doi:10.1186/s12903-016-0253-5

81. Shin J, Lee G, Kim J, Kim J, Kim S. (2017) Prevalence and clinical features of molar-incisor hypomineralization in adolescents in Yangsan. . Journal of the Korean Academy of Pediatric Dentistry ;44(2):210-219.

82. Poureslami H, Shojaiepour R, Abbaspour S, Aminizadeh M, Khademi M, Abbaspour S. (2015) Prevalence of the molar incisor hypomineralization in seven to twelve-year-old students of Kerman, Iran, in -2016. Journal of Oral Health and Oral Epidemiology. 2018;7(1):21-27.

83. Priya NS, Kumar N, Harshini T, Babu P. (2017) Prevalence, severity and clinical characteristics of Molar-incisor hypomineralization in and around Tirupati, AP among 8 to 15 years old children. . World Journal of Pharmaceutical Sciences. :256-263.

84. Buchgraber B, Kqiku L, Ebeleseder KA. (2018) Molar incisor hypomineralization: proportion and severity in primary public school children in Graz, Austria. Clin Oral Investig. Mar ;22(2):757-762. doi:10.1007/s00784-017-2150-y

85. Lago JD. (2017) Incidência da hipomineralização molar-incisivo em Araraquara e análise de fatores associados. . Universidade estadual paulista “Júlio de Mesquita Filho”; .

86. Bakkal M, Abbasoglu Z, Kargul B. (2017) The Effect of Casein Phosphopeptide-Amorphous Calcium Phosphate on Molar-Incisor Hypomineralisation:  A Pilot Study. Oral Health Prev Dent. ;15(2):163-167. doi:10.3290/j.ohpd.a37928

87. Mulic A, Cehajic E, Tveit AB, Stenhagen KR. (2017) How serious is Molar Incisor Hypomineralisation (MIH) among 8- and 9-year-old children in Bosnia-Herzegovina? A clinical study. Eur J Paediatr Dent. Jun ;18(2):153-157. doi:10.23804/ejpd.2017.18.02.12

88. Koruyucu M, Özel S, Tuna EB. (2018) Prevalence and etiology of molar-incisor hypomineralization (MIH) in the city of Istanbul. J Dent Sci. Dec ;13(4):318-328. doi:10.1016/j.jds.2018.05.002

89. Hernández M, Boj JR, Espasa E, Peretz B. (2018) First Permanent Molars and Permanent Incisors Teeth by Tooth Prevalence of Molar-Incisor-Hypomineralisation in a Group of Spanish Schoolchildren. Acta Stomatol Croat. ;52(1):4-11. doi:10.15644/asc52/1/1

90. Folayan MO, Chukwumah NM, Popoola BO, et al. (2018) Developmental defects of the enamel and its impact on the oral health quality of life of children resident in Southwest Nigeria. BMC Oral Health. Sep 27 ;18(1):160. doi:10.1186/s12903-018-0622-3

91. Folayan MO, Oyedele TA, Oziegbe E. (2018) Time expended on managing molar incisor hypomineralization in a pediatric dental clinic in Nigeria. Braz Oral Res. Aug 06 ;32:e79. doi:10.1590/1807-3107bor-2018.vol32.0079

92. Saitoh M, Nakamura Y, Hanasaki M, et al. (2018) Prevalence of molar incisor hypomineralization and regional differences throughout Japan. Environ Health Prev Med. Oct 31 ;23(1):55. doi:10.1186/s12199-018-0748-6

93. Teixeira RJPB, Andrade NS, Queiroz LCC, et al. (2018) Exploring the association between genetic and environmental factors and molar incisor hypomineralization: evidence from a twin study. Int J Paediatr Dent. Mar ;28(2):198-206. doi:10.1111/ipd.12327

94. Gambetta-Tessini K, Mariño R, Ghanim A, Calache H, Manton DJ. (2018) Carious lesion severity and demarcated hypomineralized lesions of tooth enamel in schoolchildren from Melbourne, Australia. Aust Dent J. Jun 07 ;doi:10.1111/adj.12626

95. Saber F, Waly N, Moheb D. (2018) Prevalence of molar incisor hypomineralisation in a group of Egyptian children using the short form: a cross-sectional study. . Eur Arch Paediatr Dent. ;19(5):337-345. doi:10.1007/s40368-018-0364-6

96. Rai A, Singh A, Menon I, Singh J, Rai V, Aswal GS. (2018) Molar Incisor Hypomineralization: Prevalence and Risk Factors Among 7-9 Years Old School Children in Muradnagar, Ghaziabad. Open Dent J. ;12:714-722. doi:10.2174/1745017901814010714

97. Samuel A, Asokan S, Geethapriya PR. (2017) Caries status and salivary characteristics of South Indian school children with molar incisor hypomineralization: a cross-sectional study. . Journal of Indian Association of Public Health Dentistry. ;15(2):135.

98. Padavala S SGMIHaIP. (2018) Molar Incisor Hypomineralization and Its Prevalence. Contemp Clin Dent. ;9(2):S246-S250.

99. Dantas‐Neta NB, Soares Figueiredo M, Lima CCB, et al. (2018) Factors associated with molar–incisor hypomineralisation in schoolchildren aged 8–10 years: a case–control study. International journal of paediatric dentistry. ;28(6):570-577.

100. Al-Hammad NS, Al-Dhubaiban M, Alhowaish L, Bello LL. (2018) Prevalence and clinical characteristics of molar-incisor-hypomineralization in school children in Riyadh, Saudi Arabia. Int J Med Sci Clin Invent. ;5(3)

101. Medina Ramos MC. (2019) Influencia de la hipomineralización incisivo molar en la experiencia de caries en escolares de 6 a 15 años de la isla Taquile, Puno. .

102. Namuche Maldonado LD. (2019) Prevalencia de Hipomineralización Incisivo-Molar en Niños de 8 A 12 años de la IE San Lorenzo, Chiclayo. .

103. Irigoyen-Camacho ME, Villanueva-Gutierrez T, Castano-Seiquer A, Molina-Frechero N, Zepeda-Zepeda M, Sánchez-Pérez L. (2008) Evaluating the changes in molar incisor hypomineralization prevalence: A comparison of two cross-sectional studies in two elementary schools in Mexico City between and 2017. Clin Exp Dent Res. Feb 2020;6(1):82-89. doi:10.1002/cre2.252

104. Praptiwi YH, Prayitno ND, Sukmasari S. (2019) Prevalence of Molar Incisors Hypomineralisation (MIH) in primary school children. Padjadjaran Journal of Dentistry. ;31(2):79-84.

105. Ardini YD, Ismail NN, Azni NDM, Harun NA. (2019) Molar incisor hypomineralisation: Prevalence and associated risk factors among children at the Polyclinic, Kulliyyah of Dentistry, IIUM. Materials Today: Proceedings. ;16:2351-2356.

106. Menoncin BLV, Portella PD, Ramos BLM, Assunção LRDS, de Souza JF, Menezes JVNB. (2019) Dental anxiety in schoolchildren with molar incisor hypomineralization—A population‐based cross‐sectional study. International Journal of Paediatric Dentistry. ;29(5):615-623.

107. Glodkowska N, Emerich K. (2019) Molar Incisor Hypomineralization: prevalence and severity among children from Nothern Poland. Eur J Paediatr Dent. Mar ;20(1):59-66. doi:10.23804/ejpd.2019.20.01.12

108. Santos PS, Martins-Júnior PA, Paiva SM, et al. (2019) Prevalence of self-reported dental pain and associated factors among eight- to ten-year-old Brazilian schoolchildren. PLoS One. ;14(4):e0214990. doi:10.1371/journal.pone.0214990

109. Rai PM, Jain J, Raju AS, Nair RA, Shashidhar K, Dsouza S. (2019) Prevalence of Molar Incisor Hypomineralization among School Children Aged 9 to 12 Years in Virajpet, Karnataka, India. Open Access Maced J Med Sci. Mar 30 ;7(6):1042-1046. doi:10.3889/oamjms.2019.224

110. Kılınç G, Çetin M, Köse B, Ellidokuz H. (2019) Prevalence, aetiology, and treatment of molar incisor hypomineralization in children living in Izmir City (Turkey). Int J Paediatr Dent. Nov ;29(6):775-782. doi:10.1111/ipd.12508

111. Singh PH, Bhat M. (2019) Prevalence of molar incisor hypomineralization among primary schoolchildren of three villages of Jaipur city, Rajasthan, India. . Journal of Advanced Clinical and Research Insights ;6(5):131-133.

112. Portella PD, Menoncin BLV, de Souza JF, de Menezes JVNB, Fraiz FC, Assunção LRDS. (2019) Impact of molar incisor hypomineralization on quality of life in children with early mixed dentition: A hierarchical approach. Int J Paediatr Dent. Jul ;29(4):496-506. doi:10.1111/ipd.12482

113. Reyes MRT, Fatturi AL, Menezes JVNB, Fraiz FC, Assunção LRDS, Souza JF. (2019) Demarcated opacity in primary teeth increases the prevalence of molar incisor hypomineralization. Braz Oral Res. Aug 15 ;33:e048. doi:10.1590/1807-3107bor-2019.vol33.0048

114. Goswami M, Bhushan U, Pandiyan R, Sharma S. (2019) Molar incisor hypomineralization—an emerging burden: a short study on prevalence and clinical characteristics in Central Delhi, India. . International Journal of Clinical Pediatric Dentistry. ;12(3):211.

115. Mejía JD, Restrepo M, González S, Álvarez LG, Santos-Pinto L, Escobar A. (2019) Molar Incisor Hypomineralization in Colombia: Prevalence, Severity and Associated Risk Factors. J Clin Pediatr Dent. ;43(3):185-189. doi:10.17796/1053-4625-43.3.7

116. Davenport M, Welles AD, Angelopoulou MV, et al. (2019) Prevalence of molar-incisor hypomineralization in Milwaukee, Wisconsin, USA: a pilot study. Clin Cosmet Investig Dent. ;11:109-117. doi:10.2147/CCIDE.S172736

117. Devashish SD, Agarwal SKB. (2019) Molar Incisor Hypomineralisation (MIH): Estimating prevalence and characteristics among children of Pune, Maharashtra. International Journal of Dental Research and Reviews. ;2:11.

118. Gutiérrez TV, Ortega CCB, Pérez NP, Pérez AG. (2019) Impact of Molar Incisor Hypomineralization on Oral Health-Related Quality of Life in Mexican Schoolchildren. J Clin Pediatr Dent. ;43(5):324-330. doi:10.17796/1053-4625-43.5.4

119. Głódkowska N, Emerich K. (2020) The impact of environmental air pollution on the prevalence of molar incisor hypomineralization in schoolchildren: A cross-sectional study. Adv Clin Exp Med. Dec ;29(12):1469-1477. doi:10.17219/acem/128227

120. Einollahi M, Hekmatfar S, Molaei M. (2020) Association between Molar Incisor Hypomineralization and Both Prenatal and Postnatal Factors in 8-10-Year-Old Children in Ardebil. Journal of Evolution of Medical and Dental Sciences. ;9(48):3606-3611.

121. Silva FMFD, Zhou Y, Vieira FGDF, Carvalho FMD, Costa MDC, Vieira AR. (2020) Defining the prevalence of molar incisor hypomineralization in Brazil. Pesquisa Brasileira em Odontopediatria e Clínica Integrada. ;20

122. Zafar R, Urooj A, Masood S. (2020) Frequency of Molar Incisor Hypomineralization and Associated Risk Factors–A Study from Southern Punjab, Pakistan. Biomedica. ;36(3):291.

123. Fernandes IC, Forte FDS, Sampaio FC. (2021) Molar-incisor hypomineralization (MIH), dental fluorosis, and caries in rural areas with different fluoride levels in the drinking water. Int J Paediatr Dent. Jul ;31(4):475-482. doi:10.1111/ipd.12728

124. Hamdan M, Abu-Ghefreh EA, Al-Abdallah M, Rajab LD. (2020) The prevalence and severity of molar incisor hypomineralization (MIH) among 8 year-old children in Amman, Jordan. . Egyptian Dental Journal. ;66:1989-1997.

125. Sakly EH, Amor WB, Zokkar N, Charavet C, Amor FB. (2020) Prevalence of molar incisor hypomineralisation among school children aged 7-12 years in Tunis, Tunisia. Pesquisa Brasileira em Odontopediatria e Clínica Integrada. ;20

126. Jurlina D, Uzarevic Z, Ivanisevic Z, Matijevic N, Matijevic M. (2020) Prevalence of Molar–Incisor Hypomineralization and Caries in Eight-Year-Old Children in Croatia. International Journal of Environmental Research and Public Health. ;17(7):6358.

127. Wogelius P, Viuff JH, Haubek D. (2020) Use of asthma drugs and prevalence of molar incisor hypomineralization. Int J Paediatr Dent. Nov ;30(6):734-740. doi:10.1111/ipd.12655

128. Sidhu N, Wang Y, Barrett E, Casas M. (2020) Prevalence and presentation patterns of enamel hypomineralisation (MIH and HSPM) among paediatric hospital dental patients in Toronto, Canada: a cross-sectional study. European Archives of Paediatric Dentistry. ;21:263-270.

129. Fragelli C, Barbosa TS, Bussaneli DG, Restrepo M, Cordeiro RCL, Santos-Pinto L. (2021) Aesthetic perception in children with molar incisor hypomineralization. Eur Arch Paediatr Dent. Apr ;22(2):227-234. doi:10.1007/s40368-020-00541-x

130. Tagelsir Ahmed A, Soto-Rojas AE, Dean JA, Eckert GJ, Martinez-Mier EA. (2020) Prevalence of molar-incisor hypomineralization and other enamel defects and associated sociodemographic determinants in Indiana. J Am Dent Assoc. Jul ;151(7):491-501. doi:10.1016/j.adaj.2020.02.027

131. Bahrololoomi Z, Amrollahi N, Mostafaloo N. (2020) The Prevalence and Extent of Molar-Incisor Hypo-Mineralization by Gender in a Group of Iranian Children. Iran J Public Health. Aug ;49(8):1585-1587. doi:10.18502/ijph.v49i8.3911

132. Elzein R, Chouery E, Abdel-Sater F, Bacho R, Ayoub F. (2020) Molar incisor hypomineralisation in Lebanon: prevalence and clinical characteristics. Molar incisor hypomineralisation in Lebanon: prevalence and clinical characteristics. ;21:609-616.

133. Singh R, Srivastava B, Gupta N. (2020) Prevalence and pattern of molar incisor hypomineralization in Delhi region. Journal of The Anatomical Society of India. ;69(3):150-154.

134. Ray P, Mohanty UK, Sethi D, Mahakur M, Sharma G. (2020) Prevalence and Treatment Need of Molar Incisor Hypomineralisation in 8-12 Year Old School Going Children of Cuttack, Odisha. Prevalence and Treatment Need of Molar Incisor Hypomineralisation in 8-12 Year Old School Going Children of Cuttack, Odisha. ;14(3)

135. Emmatty TB, Eby A, Joseph MJ, Bijimole J, Kavita K, Asif I. (2020) The prevalence of molar incisor hypomineralization of school children in and around Muvattupuzha, Kerala. Journal of Indian Society of Pedodontics and Preventive Dentistry. ;38(1):14-19.

136. Ganapathi A, Subramanian EMG, Jeevanandan G. (2020) Prevalence Of Molar Incisor Hypomineralization Among Pediatric Dental Patients Visiting A University Dental Hospital-A Cross Sectional Study. Int J Dentistry Oral Sci. ;S 7:24-27.

137. Bulani B, Shetiya SH, Deepti A. (2020) Prevalence of molar incisor hypomineralization amongst 8 to 11 years old government school children in Pune, Maharashtra-1 cross-sectional study. Pravara Medical Review. ;12(3):91-99.

138. Hoyte T, Kowlessar A, Ali A, Bearn D. (2020) Cross-Sectional survey to ascertain the prevalence of Molar Incisor Hypo-mineralization in the Trinidad and Tobago population. American Journal of Biomedical Science & Research. ;7(3):204-207.

139. Tseveenjav B, Furuholm J, Mulic A, et al. (2020) Estimating molar-incisor-hypomineralization among 8-year-olds based on 15-year public oral health practice-based data. Acta Odontol Scand. Oct ;78(7):535-540. doi:10.1080/00016357.2020.1751274

140. Reis PPGD. (2020) Prevalência de Hipomineralização Molar-Incisivo e sua Associação com Cárie Dentária em Escolares de Petrópolis, RJ. Universidade do Estado do Rio de Janeiro, Rio de Janeiro; .

141. Shojaeepour S, Jalali F, Shokrizadeh M, Riahi Madvar R, Torabi-Parizi M, Shojaeipour R. (2020) Assessing the prevalence of molar-incisor hypomineralization and its effects on oral health-related quality of life in children aged 8-12 years in the city of Kerman, Iran. Journal of Oral Health and Oral Epidemiology. ;9(3):143-148.

142. Dourado DG, Lima CCB, Silva RNC, et al. (2021) Molar-incisor hypomineralization in quilombola children and adolescents: A study of prevalence and associated factors. J Public Health Dent. Sep ;81(3):178-187. doi:10.1111/jphd.12429

143. Manzoor M, Manzoor R, Emmanuel BJ, Kumar M, Yeptho B, Gaikwad PG. (2021) Molar incisor hypomineralization incidence among 7–11 years old schoolchildren of two rural community of Kashmir, India. Journal of Advanced Clinical & Research Insights. ;8:101-103.

144. Alhowaish L, Baidas L, Aldhubaiban M, Bello LL, Al-Hammad N. (2021) Etiology of molar-incisor hypomineralization (MIH): A cross-sectional study of Saudi children. Children. ;8(6):466.

145. Bonzanini LIL, Arduim ADS, Lenzi TL, Hugo FN, Hilgert JB, Casagrande L. (2021) Molar-incisor hypomineralization and dental caries: A hierarchical approach in a populational-based study. Braz Dent J. ;32(6):74-82. doi:10.1590/0103-6440202104511

146. Arheiam A, Abbas S, Ballo L, Borowis E, Rashwan S, El Tantawi M. (2021) Prevalence, distribution, characteristics and associated factors of molar-incisor hypo-mineralisation among Libyan schoolchildren: A cross-sectional survey. European Archives of Paediatric Dentistry. ;22:595-601.

147. Almuallem Z, Alsuhaim A, Alqudayri A, et al. (2022) Prevalence and possible aetiological factors of molar incisor hypomineralisation in Saudi children: A cross-sectional study. The Saudi Dental Journal. ;34(1):36-44.

148. Rodríguez-Rodríguez M, Carrasco-Colmenares W, Ghanim A, Natera A, Acosta-Camargo MG. (2021) Prevalence and Distribution of Molar Incisor Hypomineralization in children receiving dental care in Caracas Metropolitan Area, Venezuela. Acta Odontol Latinoam. Aug 01 ;34(2):104-1112. doi:10.54589/aol.34/2/104

149. Osama VPRAR. (2021) Molar Incisor Hypomineralization Prevalence and Distribution in School Going Children in UAE. . J Pediatr. ;7(3):00-00.

150. Khazaei Y, Harris CP, Heinrich J, Standl M, Kühnisch J. (2021) Association Study on Nutrition in the First Year of Life and Molar-Incisor Hypomineralization (MIH)-Results from the GINIplus and LISA Birth Cohort Studies. Int J Environ Res Public Health. Oct 29 ;18(21)doi:10.3390/ijerph182111411

151. Brzovic Rajic V, Modric VE, Ivanisevic Malcic A, Gorseta K, Karlovic Z, Verzak Z. (2021) Molar Incisor Hypomineralization in Children with Intellectual Disabilities. Dent J (Basel). Feb 11 ;9(2)doi:10.3390/dj9020021

152. Ravichandra KS, Muppa R, Srikanth K, Kantipudi MJ, Ram KC. (2021) Molar Incisor Hypomineralization Prevalence in the Schoolchildren of Gannavaram Mandal, Krishna District, Andhra Pradesh, India: A Cross-sectional Study. International Journal of Clinical Pediatric Dentistry. ;14(6):737.

153. Hali H, Molania Jelodar T, Emadian M, Gohardehi S, Moosazadeh M, Salehi M. (2021) Prevalence of molar incisor hypomineralisation among school children of Sari, Iran. International Journal of Pediatrics. ;9(9):14341-14347.

154. Abdalla HE, Abuaffan AH, Kemoli AM. (2021) Molar incisor hypomineralization, prevalence, pattern and distribution in Sudanese children. BMC Oral Health. Jan 06 ;21(1):9. doi:10.1186/s12903-020-01383-1

155. Emmanuel BJ, Raja J, Shikhawat D, Yeptho B, Kumar M. (2021) Molar incisor hypomineralization incidence among 6-11-year-old schoolchildren of two rural community of Jaipur, India. Journal of Advanced Clinical and Research Insights. ;8(4):73-75.

156. Chávez Cruz KR. (2021) Asociación de hipomineralización del segundo molar primario con hipomineralización molar incisiva en escolares de una clínica pública de odontopediatría de Tijuana, Baja California. Universidad Autónoma de Baja, California; .

157. Freitas Fernandes LH, Laureano ICC, Farias L, et al. (2021) Incisor molar hypomineralization and quality of life: A population-based study with Brazilian schoolchildren. International Journal of Dentistry. ;2021

158. Farias L, Laureano ICC, Fernandes LHF, et al. (2021) Presence of molar-incisor hypomineralization is associated with dental caries in Brazilian schoolchildren. Braz Oral Res. ;35:e13. doi:10.1590/1807-3107bor-2021.vol35.0013

159. Noor Mohamed R, Basha S, Virupaxi SG, Idawara Eregowda N, Parameshwarappa P. (2021) Hypomineralized primary teeth in preterm low birth weight children and its association with molar incisor hypomineralization—A 3-year-prospective study. Children. ;8(12):1111.

160. Abdelaziz M, Krejci I, Banon J. (2022) Prevalence of Molar Incisor Hypomineralization in over 30,000 Schoolchildren in Switzerland. Journal of Clinical Pediatric Dentistry. ;46(1):1-5.

161. Mariam S, Goyal A, Dhareula A, Gauba K, Bhatia SK, Kapur A. (2022) A case-controlled investigation of risk factors associated with molar incisor hypomineralization (MIH) in 8-12 year-old children living in Chandigarh, India. Eur Arch Paediatr Dent. Feb ;23(1):97-107. doi:10.1007/s40368-021-00665-8

162. Khan A, Garg N, Mayall SS, Pathivada L, Kaur H, Yeluri R. (2022) Prevalence, Pattern, and Severity of Molar Incisor Hypomineralization in 8–12-year-old Schoolchildren of Moradabad City. International Journal of Clinical Pediatric Dentistry. ;15(2):168-174.

163. El Ghaffar A, Mahmoud S, Fouad M. (2022) Prevalence of molar incisor hypominerlization among a group of Egyptian children: A cross sectional study. Egyptian Dental Journal. ;68(1):29-37.

164. Zhang Y, Wang Y, Zhang Z, Jia J. (2023) Study on machine learning of molar incisor hypomineralization in an endemic fluorosis region in central China. Front Physiol. ;14:1088703. doi:10.3389/fphys.2023.1088703

165. Børsting T, Schuller A, van Dommelen P, et al. (2022) Maternal vitamin D status in pregnancy and molar incisor hypomineralisation and hypomineralised second primary molars in the offspring at 7-9 years of age: a longitudinal study. . Eur Arch Paediatr Dent. ;23(4):557-66. doi:10.1007/s40368-022-00712-y

166. Vanhée T, Poncelet J, Cheikh-Ali S, Bottenberg P. (2022) Prevalence, Caries, Dental Anxiety and Quality of Life in Children with MIH in Brussels, Belgium. J Clin Med. May 29 ;11(11)doi:10.3390/jcm11113065

167. Verma S, Dhinsa K, Tripathi AM, Saha S, Yadav G, Arora D. (2022) Molar Incisor Hypomineralization: Prevalence, Associated Risk Factors, Its Relation with Dental Caries and Various Enamel Surface Defects in 8-16-year-old Schoolchildren of Lucknow District. Int J Clin Pediatr Dent ;15(1):1-8. doi:10.5005/jp-journals-10005-2088

168. Ordonez-Romero I, Jijon-Granja Y, Ubilla-Mazzini W, Porro-Porro L, Alvarez-Giler G. (2019) Distribution of molar incisor hypomineralization in Ecuadorian children. Dental Hypotheses. ;10(3):65.

169. Argote Quispe DM, de Priego GPM, Leon Manco RA, Portaro CP. (2021) Molar incisor hypomineralization: Prevalence and severity in schoolchildren of Puno, Peru. J Indian Soc Pedod Prev Dent. ;39(3):246-250. doi:10.4103/jisppd.jisppd_460_20

170. Nisii F, Mazur M, De Nuccio C, et al. (2022) Prevalence of molar incisor hypomineralization among school children in Rome, Italy. Sci Rep. May 05 ;12(1):7343. doi:10.1038/s41598-022-10050-0

171. Quintero Y, Restrepo M, Rojas-Gualdrón DF, Farias AL, Santos-Pinto L. (2022) Association between hypomineralization of deciduous and molar incisor hypomineralization and dental caries. Braz Dent J. ;33(4):113-119. doi:10.1590/0103-6440202204807

172. Grieshaber A, Waltimo T, Haschemi AA, et al. (2023) Prevalence of and factors associated with molar-incisor hypomineralisation in schoolchildren in the canton of Basel-Landschaft, Switzerland. Clin Oral Investig. Feb ;27(2):871-877. doi:10.1007/s00784-022-04648-x

173. Ilczuk-Rypuła D, Zalewska M, Pietraszewska D, Dybek A, Nitecka-Buchta A, Postek-Stefańska L. (2022) Prevalence and Possible Etiological Factors of Molar-Incisor Hypomineralization (MIH) in Population of Silesian Children in Poland: A Pilot Retrospective Cohort Study. Int J Environ Res Public Health. Jul 17 ;19(14)doi:10.3390/ijerph19148697

174. Ciocan B, Săndulescu M, Luca R. (2023) Real-World Evidence on the Prevalence of Molar Incisor Hypomineralization in School Children from Bucharest, Romania. Children (Basel). Sep 16 ;10(9)doi:10.3390/children10091563

175. Al-Nerabieah Z, AlKhouli M, Dashash M. (2023) Prevalence and clinical characteristics of molar-incisor hypomineralization in Syrian children: a cross-sectional study. Sci Rep. May 26 ;13(1):8582. doi:10.1038/s41598-023-35881-3

176. Cots E, Casas M, Gregoriano M, et al. (2024) Ethnic disparities in the prevalence of molar-incisor-hypomineralisation (MIH) and caries among 6-12-year-old children in Catalonia, Spain. European journal of paediatric dentistry. ;25(3):188-199.

177. Tessari V, Ramos B, Fonseca-Souza G, et al. (2024) Prevalence of hypomineralized second primary molars and association with socioeconomic characteristics and dental caries in a Brazilian population of preschoolers: a cross-sectional study. European Archives of Paediatric Dentistry. ;25(5):767-772.

178. Chowdhury AR, Singh N, Rathore M. (2024) Molar Incisor Hypomineralization: Prevalence and Treatment Needs in 7-to 9-year-old Children of Lucknow City. International Journal of Clinical Pediatric Dentistry. ;17(7):790.

179. Afzal SH, Skaare AB, Wigen TI, Brusevold IJ. (2024) Molar-incisor hypomineralisation: severity, caries and hypersensitivity. Journal of dentistry. ;142:104881.

180. Fernandes LHF, Laureano ICC, Farias L, Prates CdC, Alencar CRBd, Cavalcanti AL. (2024) Impact of molar incisor hypomineralization on oral health-related quality of life in brazilian schoolchildren aged 8 to 10 years. Pesquisa Brasileira em Odontopediatria e Clínica Integrada. ;24:e230194.

181. Sharma S, Chauhan AS, Parthi A, Ali S, Tahseen MA. (2024) Incidence and treatment for hypomineralization of incisor and molar among school going Indian children. Bioinformation. ;20(5):575.

182. Al Jeghami R, Al-Nerabieah Z, Dashash M. (2024) Etiological Exploration of Enamel Defects: A cross-sectional study to Unravel Maternal, Prenatal, and Early Childhood Influences. ;

183. Brejawi M, Venkiteswaran A, Ergieg S, Md Sabri B. (2024) Caries experience in children with molar–incisor hypomineralisation in Fujairah, United Arab Emirates and its association with hypomineralised teeth number. European Archives of Paediatric Dentistry. ;25(2):211-216.

184. Seloğlu A, Kahvecioğlu F. (2024) Investigation of the Etiology of Molar Incisor Hypomineralization in Children Residing in Konya Province and Surrounding Areas, Türkiye. Children. ;11(11):1399.

185. Abdulla SE, Awn BH. (2024) Revalence of Molar Incisor Hypomineralization among Primary School Children in Baquba City. Diyala Journal of Medicine. ;26(2):152-162.

186. Soares LS, Fernandes EC, Santos PB. (2024) The prevalence and characteristics of molar-incisor hypomineralisation in Natal, Brazil. Pediatric Dental Journal. ;34(1):14-18.

187. Etman AM, Aboubakr RM, Alkhadragy D. (2024) Prevalence and predictors of molar-incisor hypomineralization among Egyptian children: a cross-sectional study. European Oral Research. ;58(3):120-126.

188. LOPES BC, de Souza ALBERGARDI AB, FALQUETTI BB, et al. (2024) Hipomineralização molar-incisivo: prevalência e possíveis fatores etiológicos em uma população de Araçatuba-São Paulo. Universidade Estadual Paulista Revista de Odontologia. ;53

189. LAZO HUANQUI DS. (2023) PREVALENCIA DE HIPOMINERALIZACIÓN INCISIVO MOLAR EN NIÑOS DE 6 A 11 AÑOS DE LA IE ERNESTO DE OLAZÁBAL LLOSA, PUNTA DE BOMBÓN–AREQUIPA, . 2024;

190. PREVIERO LM, GUEZIN SMV, dos Reis DOVAL G, da COSTA-SILVA CM. (2025) Prevalência e severidade da hipomineralização molar incisivo em escolares de São João da Boa Vista. Revista de Odontologia da UNESP. ;53(Especial):0-0.

191. Akyıldız BM, Sevilmiş N, Özçiftci R, Taş A, Sönmez IŞ. (2025) The Prevalence Of Molar-Incİsor Hypomineralization In Students Aged 8-12 Years In Aydın. ADO Klinik Bilimler Dergisi. ;14(3):224-232.

192. El Fitriyah R, Hartman H, Gunawan AP. (2025) DIFFERENCES IN THE PREVALENCE OF MOLAR-INCISOR HYPOMINERALIZATION BETWEEN URBAN AND RURAL AREAS. Journal of Health and Dental Sciences. ;4(3):289-300.

193. Medina Varela AF, García Pérez A, Villanueva Gutiérrez T, Mora Navarrete KA, Nieto Sánchez MP. (2024) An inverse relationship between dental fluorosis and Molar Incisor Hypomineralization in Mexican schoolchildren in an area with a high concentration of fluoride in drinking water: A cross-sectional study. Plos one. ;19(9):e0310420.

194. Baghlaf K, Bokhari GA, Aljehani FY, et al. (2024) Molar incisor hypomineralization and related risk factors among primary school children in Jeddah: a cross-sectional study. Children. ;11(10):1224.

195. Susin SC, Toscan M, de Souza VC. Prevalence of enamel development defects in very low birth weight premature infants.

196. Ortega-Luengo S, Feijóo-Garcia G, Miegimolle-Herrero M, Gallardo-López NE, Caleya-Zambrano AM. (2024) Prevalence and clinical presentation of molar incisor hypomineralisation among a population of children in the community of Madrid. BMC Oral Health. ;24(1):229.

197. Zameer M, Peeran SW, Basheer SN, Peeran SA, Naviwala GA, Birajdar SB. (2024) Molar incisor hypomineralization: Prevalence, severity and associated aetiological factors in children seeking dental care at Armed Forces Hospital Jazan, Saudi Arabia. The Saudi Dental Journal. ;36(8):1111-1116.

198. Osadolor O, Osadolor A. (2024) Prevalence of molar incisor hypomineralisation among school children in a Nigerian rural community. Ibom Medical Journal. ;17(3):442-446.
