# Supplementary material for: Diagnostic Indices for Epidemiological Assessment of Molar Incisor Hypomineralization: A Systematic Review
Source: Calcif Tissue Int. 2026 May 8;117(1):80. doi: 10.1007/s00223-026-01538-2 (PMC13156231; doi:10.1007/s00223-026-01538-2)
Supplement: Supplementary file 4 — Supplementary Material 4 [file 223_2026_1538_MOESM4_ESM.docx]

**Diagnostic Indices for Epidemiological Assessment of Molar Incisor Hypomineralization: A Systematic Review**

Marta Mazur^1^, Artnora Ndokaj^2,*^, Marta Berdzik-Janecka^3^, Irena Dus-Ilnicka^4^, Roman Ardan^5^, Sylvie Babajko^6^, Katia Jedeon^6,7^

^1^Interdisciplinary Department of Wellbeing, Health and Environmental Sustainability-BeSSA Department, Sapienza University of Rome, 02100 Rieti, Italy

^2^ Department of Oral and MaxilloFacial Sciences, Sapienza University, Via Caserta 6, 00161 Rome, Italy

^3^ **Department and Division of Paediatric Dentistry and Preclinical Dentistry Faculty of Medicine and Dentistry,** Wroclaw Medical University, ul. T. Marcinkowskiego 1, 50-368 Wroclaw, Poland

^4^ Division of General and Experimental Pathology, Department of Clinical and Experimental Pathology, Wroclaw Medical University, ul. T. Marcinkowskiego 1, 50-368 Wroclaw, Poland

^5^ Department of Economic Sciences, Koszalin University of Technology, Koszalin, Poland

^6^ Université́ Paris Cité, Université Sorbonne Paris Nord, Institut National de Santé et Recherche Médicale (INSERM), Unité Mixte de Recherche (UMR) 1333 Oral Heath, 92120 Montrouge, France

^7^ Department of Restorative Dentistry and Endodontics, Unité de Formation et de Recherche (UFR) Odontologie, Rothschild Hospital, 5 rue Santerre, 75012 Paris, France

*Corresponding author : Artnora Ndokaj, email: artnora.ndokaj@uniroma1.it

**Author contributions**

All authors contributed to the study conception and design. Material preparation, data collection and analysis were performed by Artnora Ndokaj, Roman Ardan, Marta Berdzik-Janecka and Irena Dus-Ilnicka. The first draft of the manuscript was written by Marta Mazur, Livia Ottolenghi, Sylvie Babajko and Katia Jedeon and all authors commented on previous versions of the manuscript. All authors read and approved the final manuscript.

**Abstract**

Molar incisor hypomineralization (MIH) is a qualitative developmental defect of enamel and one of the most prevalent oral health conditions in childhood. Over the last two decades, increasing attention has been given to its prevalence and etiology; however, marked heterogeneity in diagnostic criteria and indices has limited the comparability of epidemiological findings. This systematic review aimed to identify and describe the diagnostic indices used for the epidemiological assessment of MIH in the general pediatric population.

A systematic literature search was conducted in PubMed, Scopus, and Google Scholar to identify epidemiological studies published between January 2001 and December 2024 reporting MIH prevalence in children. Study selection, data extraction, and methodological quality assessment were performed independently by two reviewers in accordance with PRISMA guidelines. The review protocol was registered in PROSPERO (CRD42022345224).

A total of 198 articles were included, corresponding to 201 independent epidemiological studies, as three articles reported results from two distinct study populations. The European Academy of Paediatric Dentistry (EAPD) criteria were used in 171 studies (75.7%), representing the most widely adopted diagnostic index. Other indices, including the modified Developmental Defects of Enamel index (mDDE), Mathu-Maju and Wright, Wetzel and Reckel, MIH Severity Scoring System (MIH-SSS), Steffen criteria, and FDI criteria, were used infrequently. Studies using EAPD criteria accounted for the largest cumulative sample size and showed a descriptively aggregated MIH prevalence of 12.9%, whereas prevalence estimates varied across alternative diagnostic indices.

In conclusion, the lack of uniform diagnostic standards and nomenclature hampers global surveillance and limits the comparability of prevalence data. The adoption of MIH-specific diagnostic criteria, supported by adequate examiner calibration and transparent reporting, is essential to improve the quality, consistency, and interpretability of future epidemiological research.

**Keywords**: Molar incisor hypomineralization; Epidemiology; Prevalence; Diagnostic criteria; Systematic review
