# Supplementary material for: Diagnostic Indices for Epidemiological Assessment of Molar Incisor Hypomineralization: A Systematic Review
Source: Calcif Tissue Int. 2026 May 8;117(1):80. doi: 10.1007/s00223-026-01538-2 (PMC13156231; doi:10.1007/s00223-026-01538-2)
Supplement: Supplementary file 3 — Supplementary Material 3 [file 223_2026_1538_MOESM3_ESM.docx]

Rome, 9 March 2026

Dear Editor-in-Chief,

On behalf of all authors, I am pleased to submit our manuscript entitled “Diagnostic Indices for Epidemiological Assessment of Molar Incisor Hypomineralization: A Systematic Review” for consideration for publication in *Calcified Tissue International and Musculoskeletal Research*.

This manuscript represents a revised version of our previously submitted article (Manuscript ID: CTIN-D-26-00074). During the preparation and submission of the initial version, our research group unfortunately experienced a cybersecurity incident that affected access to several working files and supporting materials. This circumstance partially interfered with the completeness of the initial submission The original submission received detailed comments from the reviewers and was initially rejected. However, following the communication received on 2 March 2026, after discussion with the Editor-in-Chief, we were kindly invited to resubmit the manuscript in a revised format that clearly and rigorously addresses every point raised by the reviewers.

In the present resubmission, all materials have been carefully reconstructed and verified, and the manuscript has been thoroughly revised. In addition, we have addressed each point raised by the reviewers and paid particular attention to strengthening the methodological aspects of the study, as suggested by the Editorial Office.

We are sincerely grateful for this opportunity. In the present revised version, we have carefully addressed each reviewer comment point by point, and particular attention has been paid to strengthening the methodological rigor of the study, in accordance with the Editors’ recommendations. A detailed response to all reviewer comments has been provided in the accompanying document. For the convenience of the Editors and Reviewers, all modifications made in the revised manuscript are highlighted in red.

This systematic review provides an overview of the diagnostic indices applied in epidemiological studies reporting MIH prevalence worldwide between 2001 and 2024, emphasizing the need for standardized criteria to improve comparability across populations.

The manuscript is original, has not been published previously, and is not under consideration for publication elsewhere. All authors have approved the submitted version and agree to be accountable for the work.

Please note that our paper is related to the Enamel 11 Meeting.

Thank you very much for considering our resubmission.

Sincerely,

Artnora Ndokaj
