# Supplementary material for: Diagnostic Indices for Epidemiological Assessment of Molar Incisor Hypomineralization: A Systematic Review
Source: Calcif Tissue Int. 2026 May 8;117(1):80. doi: 10.1007/s00223-026-01538-2 (PMC13156231; doi:10.1007/s00223-026-01538-2)
Supplement: Supplementary file 5 — Supplementary Material 5 [file 223_2026_1538_MOESM5_ESM.docx]

Dear Editor, Dear Reviewers,

Please find our responses to your comments in italic, and all changes made to the manuscript highlighted in red text. We sincerely thank you for your time and valuable feedback, which we greatly appreciate.

COMMENTS TO THE AUTHOR:
**Reviewer #1:** The authors' responses, substantial edits lead to major improvements. The manuscript type was changed from 'Original article' to 'Review article' which is a more adequate fit for the content. The Discussion is much improved.

A very few issues remain:

Figure 1:

* On the top and right boxes several numbers are missing a subject, e.g. "389 Pubmed" consider "389 articles in Pubmed", "357 Scopus" consider "357 articles in Scopus", "3100 Google Scholar" consider "3100 articles in Google Scholar"; "849 duplicates", consider "849 duplicate articles"; "2454 excluded" consider "2454 articles excluded"; "25 not retrieved", please consider "25 articles not retrievable"; "320 excluded" consider "320 articles excluded".

*Thank you for your careful reading and for these helpful suggestions. The figure has been revised accordingly, and all labels have been modified to clearly specify the subject (i.e., “articles”) as recommended.*

* Figure 4: the icons are still difficult to recognize. How about trying using filled, color-coded squares, circles, triangles etc. The blue ocean is emphasized; try to use a lighter blue or very light grey.

*Thank you for valuable comment. We changed ocean colour to much lighter and changed to filled markers except for the dominant EAPD (to prevent parts of Europe from being completely blurred). The markers were also slightly enlarged.*

* For consistency, if using the authors names for criteria, all names should be used. The Steffen criteria were conceived and published by 3 co-authors, they all should be named or MIH TNI could be instead, but this should be consistently applied to other criteria too.

*Thank you for this valuable comment. We agree that consistency in the nomenclature of diagnostic indices is important.*

*Accordingly, we have revised the manuscript to ensure a uniform approach in the naming of all diagnostic criteria. In particular, where author-based nomenclature was used, it has been applied consistently across all indices. For clarity and standardization, we have also aligned the terminology with the most commonly used denomination for each index (e.g., MIH TNI where appropriate).*

*These revisions have been implemented throughout the manuscript to improve consistency and readability.*

* Discussion: when referring to the FDI criteria one or more references should be added.

*Thank you for this helpful comment. We agree that appropriate referencing is needed when discussing the FDI criteria. Relevant references have now been added in the Discussion section to support this statement.*

* Table S1: change 'sample' to 'subjects'.

*Thank you for this helpful suggestion. The term “sample” has been replaced with “subjects” in Table S1, as recommended.*

* Table S2: consider changing title from 'Newcastle-Ottawa scale (NOS) for cross-sectional studies' to 'Newcastle-Ottawa scale (NOS) for cross-sectional studies applied to selected MIH studies'. How are the stars defined? How is the RoB score calculated? It looks like, it is the same as the Total quality score. Is this redundant?

*Thank you for these thoughtful and constructive comments. The title of Table S2 has been revised to better reflect its specific application to MIH studies, as suggested.*

*The Newcastle-Ottawa Scale (NOS) adapted for cross-sectional studies was applied according to established criteria, with stars assigned across the domains of selection, comparability, and outcome, resulting in an overall quality score.*

*We acknowledge that the Risk of Bias (RoB) score corresponds to the total quality score. This approach was intentionally adopted to ensure consistency with commonly used NOS-based categorizations, in which predefined thresholds of the total score are used to classify studies according to their risk of bias. Rather than representing redundancy, this allows a direct interpretation of study quality in terms of risk of bias categories. To improve clarity and avoid potential misunderstanding, this has now been explicitly clarified in the table legend.*

* Table S3 is adding detailed information and strength. However, the differences, similarities, novelties etc between the indices should be explained in the main text. Under the column 'MIH specific' the meaning of 'partly' should be explained. Under the column 'Severity component', the meaning of 'limited' and 'variable' is not understandable.

*Thank you for this insightful comment. We agree that Table S3 provides detailed information, but that the key differences and conceptual aspects of the diagnostic indices should also be clearly articulated in the main text.*

*Accordingly, we have revised the Discussion section to better highlight the main similarities, differences, and distinctive features of the diagnostic indices.*

*In addition, we have clarified the meaning of the terms “partly” in the “MIH-specific” column, as well as “limited” and “variable” in the “Severity component” column, by providing explicit explanations in the table legend. These modifications were made to improve clarity and ensure accurate interpretation of the data.*

**Reviewer #2:** The manuscript addresses an important methodological issue in MIH epidemiology and provides a comprehensive overview of diagnostic indices used in prevalence studies. With further clarification of methodological aspects and minor revisions to improve transparency and readability, the manuscript could make a valuable contribution to the literature.

1. The literature search included PubMed, Scopus, and Google Scholar. While these databases cover a substantial portion of the literature, the use of Google Scholar as a primary source may introduce reproducibility limitations. Clarify how many pages or results were screened in Google Scholar. Consider acknowledging the potential limitation of Google Scholar in systematic reviews within the limitations section.

*Thank you for this important methodological comment. We agree that, although Google Scholar provides broad coverage of the literature, it may present limitations in terms of reproducibility.*

*In the present study, Google Scholar was used as a complementary source to maximize the sensitivity of the search and to identify potentially relevant studies not indexed in PubMed or Scopus. A total of 3,100 records were retrieved and screened at the title level, and duplicates were removed before further selection steps.*

*To improve transparency, this process has now been clarified in the Methods section. In addition, we have acknowledged the potential limitations related to the use of Google Scholar in the Discussion section.*

2. The Authors chose not to conduct a meta-analysis because the objective was descriptive. However, since the manuscript reports prevalence values and aggregated estimates, the Authors should clarify how they were calculated.

*Thank you for valuable comment. We added an explanation in “3.5 MIH prevalence according to diagnostic indices” section.*

3. The Discussion appropriately highlights differences between diagnostic indices. However, the manuscript would benefit from a more explicit comparison of diagnostic thresholds between indices. A brief table summarising key diagnostic characteristics could strengthen the interpretability of the results.

*Thank you for this constructive suggestion. We agree that a clear comparison of diagnostic thresholds and key characteristics is important to improve the interpretability of the findings.*

*A structured comparison of the main diagnostic indices, including their key features and conceptual differences, is already provided in Supplementary Table S3. To further address this point, we have strengthened the Discussion by explicitly highlighting the differences in diagnostic thresholds, specificity for MIH, and severity assessment among the indices.*

*We believe that the combination of the revised Discussion and Table S3 provides a comprehensive and accessible overview of the main diagnostic characteristics.*

4. Although some limitations are implied, a dedicated limitations paragraph should be added to the Discussion. Important points include potential bias introduced by Google Scholar searches, absence of meta-analysis or possible publication bias.

*Thank you for this valuable suggestion. We agree that a clearer and more explicit presentation of the study limitations would improve the transparency of the manuscript.*

*Accordingly, a dedicated paragraph has now been added to the Discussion section outlining the main limitations of the study. These include the potential limitations related to the use of Google Scholar as a search source, as well as the descriptive nature of the review, which did not include a meta-analysis and may be subject to publication bias.*

5. Table 1 is informative but would benefit from clear definition of "Other" diagnostic indices and explanation of how the mean quality score was calculated.
*Thank you for this helpful comment. We agree that further clarification would improve the interpretability of Table 1.*

*Accordingly, we have now clarified the meaning of the “Other” category, specifying that it includes diagnostic approaches not corresponding to established or widely used indices. In addition, we have provided an explicit explanation of how the mean methodological quality score was calculated, indicating that it represents the average Newcastle–Ottawa Scale score across studies within each diagnostic category.*

*These clarifications have been added to the manuscript.*
